# Comprehensive Analysis of Signal Peptides in *Saccharomyces cerevisiae* Reveals Features for Efficient Secretion

**DOI:** 10.1002/advs.202203433

**Published:** 2022-12-07

**Authors:** Songlyu Xue, Xiufang Liu, Yuyang Pan, Chufan Xiao, Yunzi Feng, Lin Zheng, Mouming Zhao, Mingtao Huang

**Affiliations:** ^1^ School of Food Science and Engineering South China University of Technology Guangzhou 510641 China; ^2^ Guangdong Food Green Processing and Nutrition Regulation Technologies Research Center Guangzhou 510650 China

**Keywords:** accessory protein influence, evolutionary relationship, protein secretion, sequence analysis, signal peptide

## Abstract

Signal peptides (SPs) are N‐terminus sequences on the nascent polypeptide for protein export or localization delivery, which are essential for maintaining cell function. SPs are also employed as a key element for industrial production of secreted recombinant proteins. Yet, detailed information and rules about SPs and their cellular interactions are still not well understood. Here, systematic bioinformatics analysis and secretion capacity measurement of genome‐wide SPs from the model organism *Saccharomyces cerevisiae* is performed. Several key features of SPs, including region properties, consensus motifs, evolutionary relationships, codon bias, e.g., are successfully revealed. Diverse cell metabolism can be trigged by using different SPs for heterologous protein secretion. Influences on SPs with different properties by chaperones can cause different secretory efficiencies. Protein secretion by the SP NCW2 in *SEC72* deletion strain is 10 times than the control. These findings provide insights into the properties and functions of SPs and contribute to both fundamental research and industrial application.

## Graphical abstract

Signal peptides (SPs) are essential for protein export or localization delivery to maintain cell function. Yet, detailed information and rules about SPs and their cellular interactions are still not well understood. Here, the authors present a comprehensively analysis of SPs in *S. cerevisiae*. Key features of SPs, secretion efficiency of different SPs and influences by accessory proteins are revealed.

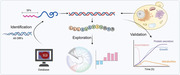

## 1 Introduction

A signal peptide (SP) is a short peptide on the N‐terminal of secretory or membrane proteins first found in the 1970s,^[^ ^1^, ^2^ ^]^ which acts as a recognized element in nascent polypeptides for targeting and transportation to cell membrane or endoplasmic reticulum (ER) through secretory pathways. SP is removed by signal peptidase and thus protein can be further folded properly and flow toward its destination.^[^ ^3^ ^]^ SPs are important for cell function. For instance, 26 human diseases have been identified as being associated with SP mutations, which indicates the necessity in study of SP mechanisms.^[^ ^4^ ^]^ Yet, many detailed processes, for example, whether and how SPs with different sequence motifs encounter different processes, remain unclear. A recent cryo‐electron microscopy study explained how structures of the Sec61 channel assist SPs in translocating,^[^ ^5^ ^]^ but which SPs are targeted and translocated closely with the Sec system have not been reported. *Saccharomyces cerevisiae*, as a model organism, has been used to identify and reveal many biological processes, and provide useful information that can be expanded to other organisms.^[^ ^6^, ^7^, ^8^ ^]^ However, to date, analysis of SPs in *S. cerevisiae* has not been comprehensive.

Recombinant protein production has become one of the most important industries in the field of modern biotechnology.^[^ ^9^ ^]^ Secretory expression is preferred in recombinant protein production, as it assures proper translational modification of recombinant proteins and simplifies downstream purification processes.^[^ ^10^, ^11^, ^12^ ^]^ Of the various engineering elements for improving protein secretion,^[^ ^13^ ^]^ SPs play a crucial role in guiding recombinant polypeptide translocation into membrane of ER by co‐ or post‐translational pathway, preventing early polypeptide aggregation.^[^ ^14^, ^15^ ^]^ Hence, constructing a good performing SP would allow for efficient secretion. Yet, limited understanding of the mechanisms hinders rational design of SPs for efficient protein secretion.^[^ ^16^ ^]^ Therefore, exploration of SP mechanisms also contributes to facilitating industrial biotechnology.

Here, we made a comprehensive study of genome‐wide SPs from *S. cerevisiae*. Systematic bioinformatics analysis and secretion capacity measurement of all SPs were performed. Meanwhile, various physiological traits of cells with different SPs for protein secretion were determined. The impact of secretion by several accessory proteins on SPs was also revealed. These findings expand our knowledge of the fundamental characterizations of SPs and improve our understanding of the underlying mechanisms of protein secretion. Furthermore, the outcomes also provide useful information and general guidelines for the selection and optimization of SPs for efficient protein secretion.

## 2 Results

### 2.1 SP Sequence Analysis

**Figure 1:**
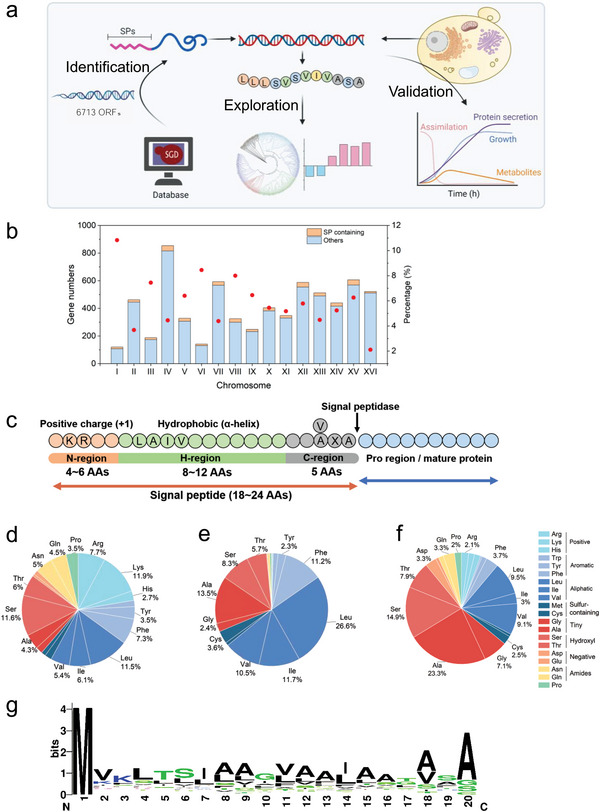
Identification and characterization of SPs. a) Schematic workflow for SPs analysis in this study. b) Distribution of SP‐containing genes on chromosomes of *S. cerevisiae*. c) General sequence feature of SPs in *S. cerevisiae*. Amino acid proportion of SPs on d) N‐region, e) H‐region, and f) C‐region; all amino acids were showed on pie charts but only those proportion >2% were labeled. g) Typical sequence conservation of SPs with 20 AAs.

To unveil the underlying mechanisms of SPs in *Saccharomyces cerevisiae*, we first accessed the whole genomic sequences (containing 6713 open reading frames (ORFs)) from the *Saccharomyces* Genome Database (SGD) and predicted potential SP sequences by SignalP5.0^[^ ^17^ ^]^ (**Figure** **1**a). Totally, 352 SPs were identified and showed distinguished probability from non‐SPs (Figure 1a; and Table S1, Supporting Information). The distribution of SP containing genes on different chromosomes are shown as well. The percentage of SP‐containing genes on most chromosomes fell in the range of ≈4–8%, besides Chr I (10.9%) and Chr XVI (2.1%) (Figure 1b; and Figure S1b, Supporting Information).

We found that SPs of *S. cerevisiae* are commonly ≈18–24 amino acids (AAs) in length (Figure S1c, Supporting Information), which is in agreement with that of eukaryotes reported previously.^[^ ^18^ ^]^ Three regions of SPs have different lengths (Figure S1d, Supporting Information). The N‐region varies between ≈2 and 10 AAs, but some extremely long N‐regions (up to 20 AAs) were also found. By contrast, the H‐region and the C‐region show relatively restricted numbers in AA residues (≈8–12 AAs for the H‐region and ≈3–8 polar and uncharged AAs for the C‐region). It is worth noting that 64.38% of the C‐region is composed of only 5 AAs, which implies that a five‐residue distance to ER membrane may be the most efficient catalytic site for a signal peptidase. Furthermore, three regions of SPs have different AA components, besides diverse AA length. The SP features of *S. cerevisiae* was showed in Figure 1c. The N‐region has a higher proportion of positive AAs (22.37%) than the other two regions, with nearly all SPs contains a positive charge AA in the N‐region on average. These positive AAs are predominantly arginine and lysine (87.86% of positive AAs) (Figure 1d). The H‐region mainly is constituted of hydrophobic AAs, especially leucine, which accounts for 26.59% of all AAs in the H‐region (Figure 1e). The C‐region has more polar and uncharged AAs, for example, the alanine constitutes 23.26% (Figure 1f). The Weblogo analysis revealed conservation sites within SPs, especially the Ala at (−3, −1) position of the C‐region was confirmed Ala‐x‐Ala (AXA) motif again in *S. cerevisiae* ^[^ ^18^ ^]^ (Figure 1g; and Figure S2, Supporting Information). Based on our analysis, the *S. cerevisiae* has 19.9% of AXA motif in SPs. Meanwhile, we also found 21.1% VXA motif in *S. cerevisiae*. To explore whether the AXA and VXA motifs present in other yeast, we analyzed all potential SPs of *Pichia pastoris* (*Komagataella phaffii*) and found similar motif proportions (22.0% AXA and 22.8% VXA) to *S. cerevisiae*.

**Figure 2:**
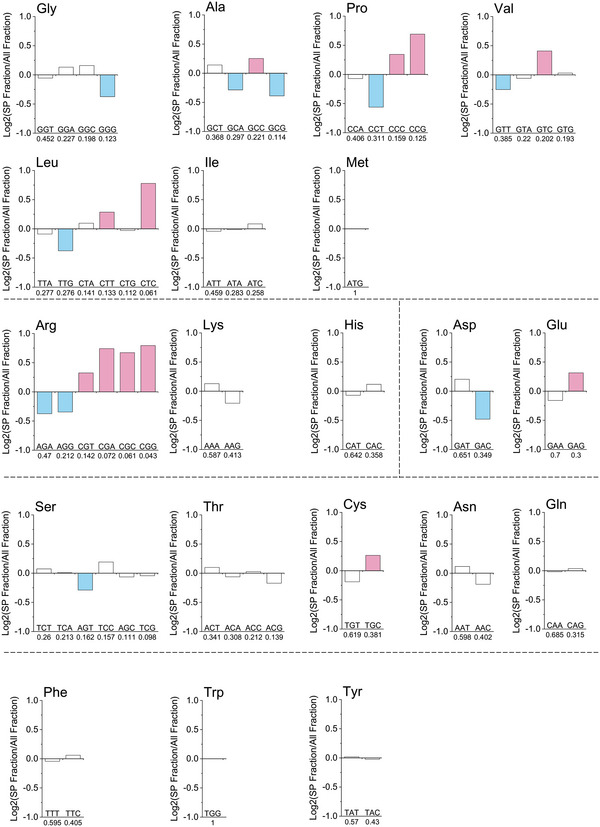
Synonymous codons proportion of 352 SP sequences compared to all 6713 ORF sequences. SP fraction represents the proportion of synonymous codons in 352 SP sequences, and All fraction represents the proportion of synonymous codons in the all full‐length ORF sequences. X axis indicates codon bias usage in yeast.

Compared with the codon bias of all ORF sequences in *S. cerevisiae*, the codon bias of SP sequences differed in some amino acids, including Arg, Pro, Leu, and Val. These amino acids tend to use rare synonymous codons in SP sequences (**Figure** **2**; and Table S2, Supporting Information).

### 2.2 Heterologous Protein Secretion Mediated by SPs

**Figure 3:**
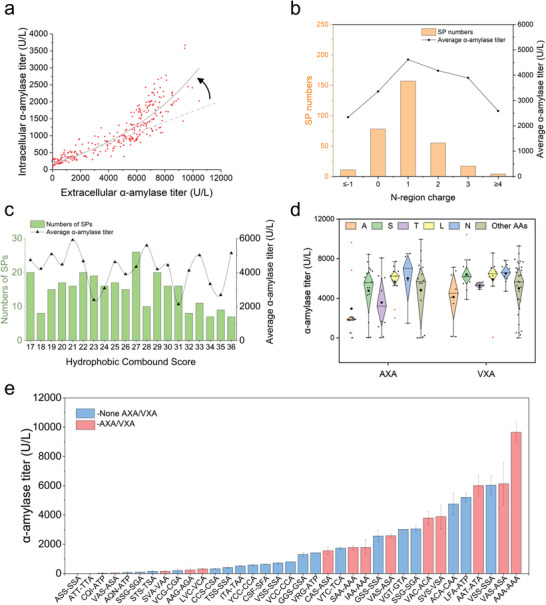
Identified SPs showed different performances in guiding heterologous protein secretion. a) Totally, 322 SPs were tested for *α*‐amylase secretion; both intracellular and extracellular level were quantified. b) Average *α*‐amylase secretion and numbers of SPs with different N‐region charges. c) Average *α*‐amylase secretion and numbers of SPs with different hydrophobicity scores. d) *α*‐amylase secretion of SPs with different (−3, −1) motifs. X indicated any amino acids in AXA or VXA motifs. e) *α*‐amylase secretion of 36 SPs with new predicted (−3, −1) motifs when fusion with *α*‐amylase cassette. A motif before the hyphen represented predicted (−3, −1) motif on SP‐native protein, and motif after the hyphen represented new predicted (−3, −1) motif on SP‐amylase sequence; data shown are mean values ±SDs of duplicates.

To evaluate the secretion capacity of the identified SPs, all 322 SPs (30 duplicate SPs were removed from 352 SPs) were fused with a model protein *α*‐amylase^[^ ^19^ ^]^ for guiding its secretion in yeast. The SPs showed different performances in guiding heterologous protein secretion (**Figure** **3**a). Interestingly, the intracellular *α*‐amylase retention increased with increased secreted *α*‐amylase, which was mediated by more efficient SPs. This indicates that downstream cellular processes in protein secretory pathway become bottlenecks when efficient SPs are used for leading secretion. Based on these *α*‐amylase secretion results, SPs were analyzed and shown in more detail. First, SPs with a certain length in subregions were more efficient in guiding secretion, such as the N‐region with 5AAs, the H‐region with ≈8–9 AAs and the C‐region with 5AAs (Figure S3a–c, Supporting Information). Second, the N‐region with a positive charge AA had the highest *α*‐amylase secretion (Figure 3b). As the hydrophobic H‐region has been reported to interact with the hydrophobic domain of signal recognition particle (SRP) or other chaperone proteins before targeting ER,^[^ ^20^ ^]^ the hydrophobic compound score of the H‐region was calculated (Figure 3c). Interestingly, secretion of SPs with a score of 23 or 24 decreased. We randomly selected 5 SPs within this hydrophobic interval and changed their hydrophobic property by deleting one amino acid residue. Four of these 5 modified SPs improved *α*‐amylase secretion significantly (Figure S3d, Supporting Information). The C‐region composed of 5 AAs took up to 60.31% and showed higher *α*‐amylase secretion, which implies a suitable length for efficient cleavage. The (−3, −1) rule^[^ ^21^ ^]^ for amino acid constituents on the C‐terminus was reported efficient for SP cleavage. This rule was also confirmed by SPs with AXA/VXA motifs having higher *α*‐amylase secretion (Figure S3e, Supporting Information). The AXA/VXA motifs with different AAs in the middle varied in *α*‐amylase production (Figure 3d). Furthermore, cleavage efficiency may be affected by the later sequence of mature protein or proregion, besides determination by preceding motifs. New sequences (322 SPs followed by the *α*‐amylase coding region, which replaced the original gene coding region associated with SPs) were predicted for cleavage sites and 36 of these new sequences were found with altered cutting sites (Figure 3e). We inferred that these 36 SPs had inefficient cleavage motifs. Therefore, they could be easily affected by following sequences, which resulted in more efficient recognized sites (such as AXA/VXA) for signal peptidase. Indeed, the average secretion (2105 U L^−1^) of *α*‐amylase led by these 36 SPs (Figure 3e) was just half of the control (average of all SPs, 4106 U L^−1^). And SPs with AXA/VXA motif on (−3, −1) position as their new prediction sites had double the *α*‐amylase secreted titer than the others (Figure S3f, Supporting Information). These findings not only emphasize the importance of efficient cleavage site of AXA/VXA on SPs, but also provide a useful strategy for efficient matching on SP cutting sites by considering subsequent protein/proregion sequences.

Regarding SPs, our findings revealed several rules for efficient protein secretion. We also noticed that there are 13 SPs followed by a pro‐peptide region, and the well‐known *α*‐factor leader is one of these 13 SPs. We tested these 13 SPs together with their pro‐regions for guiding *α*‐amylase secretion and found 7 pre‐pro peptides were better than the *α*‐factor leader (Figure S3g, Supporting Information), which has been extensively used for heterologous protein expression cross different hosts.^[^ ^22^, ^23^, ^24^ ^]^ This result indicated other SPs were worth further study to explore their potential for guiding protein secretion.

As mentioned above, several amino acids use rare codons in SPs. To reveal whether the secretion efficiency of SPs was affected by rare codons bias, several SPs were selected for validation. These SPs with codon optimization and rare codons substitution were compared with origin SPs in terms of protein secretion and cell stress. We found that many SPs with increased rare codon proportion did not increase *α*‐amylase secretion and had similar intracellular percentage, but the ROS level of strains reduced when rare codons increased (Figure S4a–d, Supporting Information). A reduced ROS level suggests that rare codon bias of SPs may bring less stress to cells. This can be considered as a positive effect of codon bias.

Secretion capacities of all SPs in *S. cerevisiae* were determined by expression of *α*‐amylase. The top 11 SPs with superior performances and 3 poor‐capacity SPs in expression of *α*‐amylase together with the *α*‐factor leader were tested for secretion of another protein, lipase. Lipase secretion results were consistent with those of *α*‐amylase, no lipase activity was detected by using the 3 poor‐capacity SPs, and all the top11 SPs had higher lipase secretion than when using the *α*‐factor leader (Figure S5, Supporting Information). These results showed that our findings not only worked for *α*‐amylase but are also likely applicable for other proteins.

### 2.3 Strains Show Different Physiological Traits When Using Different SPs for Secretion

**Figure 4:**
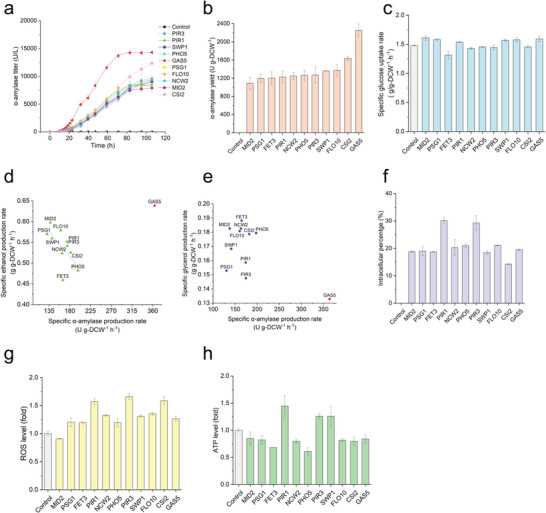
Physiological traits of super‐secreted SP strains. a) *α*‐amylase secretion level. b) Extracellular *α*‐amylase yield at the end of fermentation. c) Specific glucose uptake rate. d) specific ethanol production rate versus specific amylase production rate. e) specific ethanol production rate versus specific amylase production rate. f) Intracellular *α*‐amylase retention at the end of fermentation. g) ROS level at the exponential phase (OD_600_≈1). h) and ATP level at the exponential phase (OD_600_≈1). A strain transformed with the empty plasmid was used as control. Strains were cultivated in SD‐2 × SCAA medium with initial OD_600_ = 0.01, 30 °C and 200 rpm. Data shown are mean values ±SDs of duplicates.

To better understand cell responses by using different SPs, quantitative phenotypic information was detected for SP strains with the top 11 highest secretion capacities (super‐secreted group) in batch fermentation (**Table** **1**). GAS5 strain and CSI2 strain had excellent *α*‐amylase production rates compared with the others (**Figure** **4**a,b), GAS5 showed efficient production ability and ended secretion earlier (at 80 h), and CSI2 strain maintained secretion at the late stage (after 80 h) when other strains reached a plateau. Compared with the control (without *α*‐amylase expression), most of the strains had lower specific growth rates, but specific glucose uptake rates did not change in a consistent trend (Figure 4c). This indicated protein secretion imposed general stress on strain growth, but glucose assimilation in the super‐secreted group was more likely associated with secondary cellular regulation caused by different SPs. We found MID2 strain had the highest glucose uptake rate but achieved the lowest *α*‐amylase secretion yield within the super‐secreted group (Figure 4b,c). Also, we found that maximum specific growth was positively correlated with the specific ethanol production rate, but negatively correlated with the glycerol production rate (Figure S6a,b, Supporting Information). The GAS5 strain was quite similar to the control strain regarding ethanol production and glycerol production (Figure S6a,b, Supporting Information). The specific *α*‐amylase production rate was negatively correlated with the specific ethanol production rate, except for GAS5 (Figure 4d). Compared with other strains, GAS5 also differed in the relationship between the specific *α*‐amylase production rate and the glycerol production rate (Figure 4e).

**Table 1:** Physiological traits of different SP strains

Strain^a)^	µ_max_ ^b)^	*r*_S_ ^c)^	*r*_E_ ^d)^	*r*_G_ ^e)^	*r*_A_ ^f)^	*r*_P_ ^g)^	*r*_Amy_ ^h)^
NC	0.262±0.001	1.478±0.010	0.622±0.022	0.131±0.004	0.057±0	0.014±0	ND
PSG1	0.233±0.007	1.582±0.017	0.570±0.008	0.153±0.001	0.062±0.002	0.013±0.001	130.62±2.10
MID2	0.221±0.002	1.611±0.042	0.598±0.015	0.183±0.004	0.056±0.001	0.013±0	137.86±3.99
SWP1	0.235±0.001	1.569±0.013	0.559±0.013	0.168±0.006	0.056±0	0.013±0.001	141.02±8.00
FLO10	0.221±0.005	1.578±0.037	0.579±0.011	0.181±0.004	0.056±0.001	0.012±0	160.24±7.12
NCW2	0.212±0.003	1.431±0.018	0.524±0.018	0.183±0.014	0.051±0.001	0.011±0	163.29±14.51
FET3	0.203±0.006	1.314±0.063	0.459±0.003	0.188±0.003	0.051±0.001	0.010±0	164.74±7.03
PIR1	0.234±0.004	1.540±0.009	0.551±0.002	0.159±0	0.060±0	0.014±0	173.96±6.80
PIR3	0.231±0.003	1.444±0.042	0.541±0.020	0.148±0.005	0.057±0.002	0.013±0	174.49±10.19
CSI2	0.220±0.002	1.455±0.017	0.525±0	0.179±0.001	0.058±0.002	0.010±0.001	181.91±8.24
PHO5	0.206±0.008	1.455±0.008	0.482±0.007	0.179±0.004	0.054±0.001	0.012±0.001	197.61±11.08
GAS5	0.257±0	1.593±0.047	0.639±0.015	0.133±0.006	0.061±0.002	0.012±0.001	364.11±32.66

^a)^ Data shown are mean ± standard deviation of duplicates. ND: No detected;

^b)^ µ_max_: maximum specific growth rate (h^−1^) on glucose;

^c)^ *r*_S_: specific glucose uptake rate (g g‐DCW^−1^ h^−1^);

^d)^ *r*_E_: specific ethanol production rate (g g‐DCW^−1^ h^−1^);

^e)^ *r*_G_: specific glycerol production rate (g g‐DCW^−1^ h^−1^);

^f)^ *r*_A_: specific acetate production rate (g g‐DCW^−1^ h^−1^);

^g)^ *r*_P_: specific pyruvate production rate (g g‐DCW^−1^ h^−1^);

^h)^ *r*_Amy_: specific *α*‐amylase production rate (U g‐DCW^−1^ h^−1^).

Meanwhile, intracellular *α*‐amylase retention varied among strains in the super‐secreted group (Figure 4f). Strains PIR3 and PIR1 had higher retention (up to 30%), and CSI2 strain had much lower retention (14.2%). Nevertheless, PIR3, PIR1, and CSI2 had a higher ROS level than the other strains (Figure 4g). ATP pool in PIR3 and PIR1 strains also increased (Figure 4h). Interestingly, PHO5 and FET3, two slow‐growth strains, kept relatively low ATP levels, but fast growth strain GAS5 was not rich in ATP pool. These findings suggest that secretion processes associated with different SPs may interact with a diverse cellular metabolism, which may be involved in different chaperones or factors and result in different cellular responses.

### 2.4 Gene Ontology (GO) Analysis of SP Containing Genes

**Figure 5:**
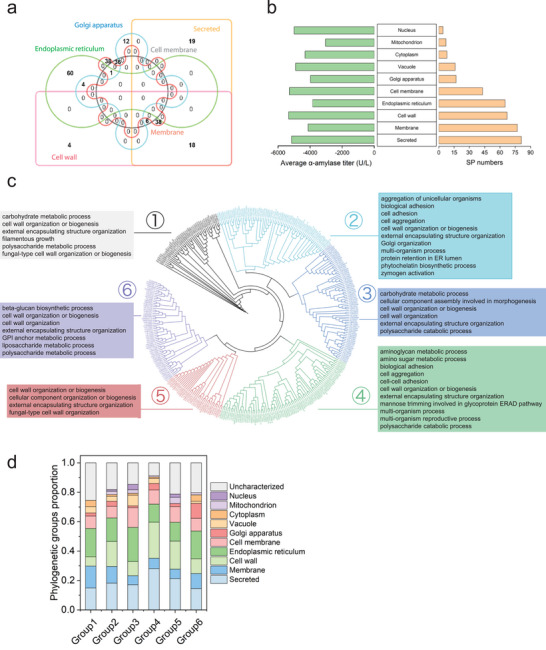
GO analysis and phylogenetic analysis revealed evolutionary relationship and function fitness of SPs. a) Six main subcellular locations of 256 SP‐containing proteins based on the UniProt annotation. b) Average extracellular *α*‐amylase secretion and numbers of 256 SPs in different categories, which were classified by UniProt‐annotated subcellular localization of SP‐containing proteins. c) Phylogenetic tree construction only based on SP sequences; six groups were classified. SP‐containing genes in each group were used for GO term enrichment analysis. d) Subcellular location proportion of SP‐containing proteins in each group.

To deeply explore physiological information through SP sequences, we first classified 256 proteins with verified subcellular localization out of the 352 SPs based on their location. Most of the yeast proteins with SPs were secreted or located at membrane, cell wall, ER, and cell membrane (**Figure** **5**a). SPs from proteins with extracellular location (secreted, cell wall, and cell membrane) showed stronger abilities for guiding amylase secretion (Figure 5b). This result reflects that SPs have evolved suitable sequences to support the needs of relevant proteins. For instance, extracellular location proteins require efficient secretion. GO analysis was performed on SP‐containing genes in *S. cerevisiae* (Figure S7a–c; and Table S3, Supporting Information). Many GO terms were related to the external encapsulating structure, fungal‐type cell wall, extracellular region, anchored component of membrane, etc., which were consistent with extracellular function and location. As mentioned above, SPs develop their sequences through the natural evolution process. We performed phylogenetic tree analysis of SPs based only on their sequences, and they could be classified into 6 groups (Figure 5c,d). The GO terms of the 6 phylogenetic groups showed different biological process abundance. Although these 6 groups were classified only according to SP sequences, GO analysis of each group revealed functional differentiation (Figure 5c). Group five showed a lower external sublocation proportion than group four, but showed abundance at external physiological processed such as fungal‐type cell wall organization, cell wall organization, or biogenesis and external encapsulating structure organization (Figure S8, Supporting Information). These results mean proteins in group five had evolved concentrated SP sequences. Group six had more Golgi apparatus proteins. And GO terms Golgi to endosome transport and GPI anchor metabolic progress were also enriched. These results indicate evolution of SPs is closely linked with native conjoint proteins to fit protein function.

More evidence about SP evolution could be found from paralog gene groups with SP mutation and each SP pair showed different secretion capacity (Figure S9, Supporting Information). For example, CCW12 and CCW22 had different predicted cleavage sites on the C‐region. The −1 position of CCW12 mutating to Ala from Ser resulted in CCW22, which showed higher *α*‐amylase secretion. When the last 2 residues on CCW12 SP and Val/Ile in H‐region were removed, named CCW12*, we expectedly found a similar *α*‐amylase secretion level of CCW12* to CCW22 SP (Figure S10, Supporting Information). We also found rapid‐evolving PAU family with 24 duplicated members, which have evolved to 21 precursor proteins (the other 3 proteins had N‐region deletion or substitution) with 5 SPs. Interestingly, 4 out of these 5 SPs from 20 members had the common AXA/VXA motif in (−3, −1) and *α*‐amylase titer was higher than that of the one with IAA motif.

### 2.5 Loss of Accessory Proteins Has an Influence on Secretion

To validate whether protein secretion changed using different SPs, if loss of accessory proteins, 8 genes (*SND1, SND2, SND3, STE24, SPC1, SEC72, APJ1, JJJ3*) were deleted and shown impact on *α*‐amylase secretion led by super‐secreted group SPs and the *α*‐factor leader, and we did find secretion changes in deletion strains. Snd1, Snd2, and Snd3 were recently confirmed as new proteins in the SRP‐independent pathway for ER targeting when the signal recognition particle (SRP) pathway and guided entry of tail‐anchored proteins (GET) pathway lose function.^[^ ^25^ ^]^ When *SND3* was deleted, nearly all SP strains decreased secretion, except the CSI2 strain (Figure S11a, Supporting Information). Unlike *SND3*, deletion of *SND1* or *SND2* had no effect on *α*‐amylase secretion. This finding suggests that Snd3 might be involved in a novel pathway, which affects nascent poly peptides targeting ER. Ste24 has been reported as an ER membrane protein to remove nascent polypeptide clogging in translocation.^[^ ^26^, ^27^ ^]^ Our findings showed all SP strains reduced secretion after deletion of *STE24*, and the FET3 strain even completely lost secretion capacity (Figure S11b, Supporting Information), which meant the SP FET3 relied more heavily on Ste24 medicated translocation than other SPs. Our findings demonstrated that protein secretion levels could be affected by different SPs, if lack of some accessory proteins.

**Figure 6:**
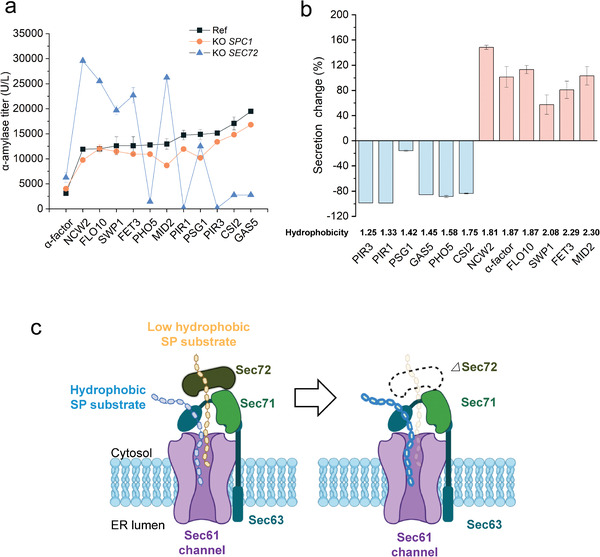
Influences on heterologous protein secretion by accessory proteins linked to SPs with different properties. a) *α*‐amylase secretion titer in the ref, △*SPC1* and △*SEC72* strains when using different SPs. The *α*‐factor SP was used as a reference to compared with other 11 super‐secreted SPs. b) Hydrophobicity of SPs determined *α*‐amylase secretion change tendency (increase or decrease) in the △*SEC72* strain. The secretion change was calculated as follow: (amylase secretion by the △*SEC72* strain – amylase secretion by the ref strain)/(amylase secretion by the ref strain) × 100%. Ref: strain MSBP003 was used as the control strain. Data shown are mean values ±SDs of duplicates. c) SP substrates translocation schematic diagram after SEC72 deletion. Dotted line represent deletion of SEC72 protein. Deeper colors represent the promotion of hydrophobic SP substrate translocation, while light color represent the depression of low hydrophobic SP substrate.

Spc1 is an evolutionarily conserved subunit of signal peptidase complex but dispensable for signal peptidase activity, whose molecular function is still unclear.^[^ ^28^, ^29^ ^]^ When we knocked out Spc1, strains with PSG1 and MID2 had relatively greater decrease in *α*‐amylase secretion (**Figure** **6**a). We further compared the (−3, −1) motif of SPs and surprisingly found only −2 position of PSG1 and MID2 SP sequences are both basic amino acids (Figure S12, Supporting Information). Interestingly, Sin1‐Spc1, an interaction in the MAPK pathway, have been identified as clustered basic residues in Sin1 recognized by conserved acidic residues (Asp‐304 and Asp‐307) of Spc1.^[^ ^30^ ^]^ This hints that Spc1 may play an exact role in signal peptidase complex for substrates with specific motifs, and the amino acid on −2 position in (−3, −1) motif may contribute to SPs processing.

Sec72 is a component of the Sec62/Sec63 complex involved in post‐translational translocation of proteins into ER.^[^ ^31^, ^32^ ^]^ Almost all SP strains with *SEC72* deletion drastically altered *α*‐amylase secretory capacity (Figure 6a). The *α*‐amylase secretion by the SP NCW2 in the *SEC72* deletion strain was more than 10 times higher compared with that of the control strain using the *α*‐factor leader. Cotranslational translocation involves an interaction of precursor and SRP, which allows hydrophobic SP sequences recognized by SRP,^[^ ^33^ ^]^ while SPs with low hydrophobic core sequences mainly depend on the post‐translational translocation pathway involving interaction of HSP70 and Sec72.^[^ ^34^, ^35^ ^]^ Hence, we calculated hydrophobic compound scores of SPs. Low hydrophobic SPs (scores≤1.75) had impaired secretion capacity in the *SEC72* deletion strain and *α*‐amylase secretion was even abolished entirely in PIR1 and PIR3 strains (Figure 6b; and Table S4, Supporting Information). This result is consistent with a reported study in which sec72 helped in recognizing and translocating SPs with low hydrophobicity, and our study found different affects according to SP sequence bioinformation. In addition, strains with high hydrophobic SP (scores≥1.81) had much better performance on expressing *α*‐amylase, which showed loss of Sec72 increased protein secretion with high hydrophobic SPs and SP composition is crucial in the Sec system.

## 3 Discussion

SPs are one of the major components in secretory systems that assist proper protein localization.^[^ ^15^ ^]^ Improving our understanding of SPs is of importance to reveal cell state from the point of view of secreted proteins^[^ ^36^ ^]^ and modify cellular networks for better performance.^[^ ^37^ ^]^ Here, we performed a genome wide analysis of SPs in *S. cerevisiae*, and several fundamental mechanisms for efficient secretion were revealed and validated, which have not before been comprehensively reported. The distributions of SP‐containing genes on most chromosomes range between 4% and 8%, except for Chr I with 10.9% and Chr XVI with 2.1%. This may provide evolutionary clues about SP‐containing genes on different yeast chromosomes. Most SPs in yeast have a length of 18–24 AAs. Three regions of SPs have a distinct length. Yeast SPs have rich positive charge AAs in the N‐region, which contributes to efficient translocation. A loss of the N‐region positive charge impaired translocation of small secretory preproteins and caused cell dysfunction.^[^ ^38^ ^]^ Two‐thirds of the C‐region is composed of only 5 AAs, which indicates a suitable distance for processing by signal peptidases. The typical AXA/VXA motifs were found in the C‐region of SPs in yeasts. This (−3, −1) rule/motif, has also been reported in other species, but the proportions were different in various species. The occurrence of AXA motif through analysis of 1877 eukaryotic, 168 Gram‐positive, and 307 Gram‐negative SPs was 14.5%, 47.0% and 58.9%, respectively.^[^ ^18^ ^]^ The proportion of AXA motif in *S. cerevisiae* (19.9%) was higher than that of eukaryotes, and less than that of prokaryotes. This also provided clues to show the evolutionary status of yeast as single cell eukaryotic microorganisms, which were between higher eukaryotes and prokaryotes. The yeast *S. cerevisiae* was used as a model organism for studying diseases linking with intracellular trafficking or secretion and screening potential drugs.^[^ ^39^, ^40^, ^41^, ^42^ ^]^ More understanding of SPs may contribute to design of a comprehensive yeast model for studying biological processes and finding treatments of relevant diseases.

SPs possess their sequences through long‐term evolution. Based on GO analysis results, evolution of SPs is closely linked with relevant proteins to fit protein function. The *α*‐amylase secretion level by yeast SPs crossed four orders of magnitude (0–10^4^ U L^−1^), which revealed diverse capacities of SPs in guiding protein secretion. In case of using efficient SPs, downstream cellular processes in secretory pathways become limited, as intracellular retention raises with increased secreted *α*‐amylase. Heterologous protein secretion was increased by engineering yeast with targets in ER to Golgi vesicle trafficking, Golgi to plasma membrane vesicle trafficking and trafficking between different subcellular organelles.^[^ ^43^, ^44^, ^45^, ^46^ ^]^ A modular pathway optimization method^[^ ^47^ ^]^ can be applied to relieve bottleneck and promote secretion for efficient SPs. Seven SPs together with their original pro‐region showed better secretion than the widely used *α*‐factor leader. Previously, efficient secretory leaders were obtained through directed evolution of the *α*‐factor leader.^[^ ^16^, ^48^ ^]^ Our result suggests SPs in yeast are worth further study to explore their potential for guiding protein secretion and provide useful information in rational design of secretory leaders.^[^ ^49^, ^50^ ^]^ Besides preceding motifs in the C region, SP cleavage efficiency may be affected by subsequent sequences after the C‐terminus of SP. Therefore, inclusion of AAs after peptidase cutting site in SP design should be beneficial for efficient secretion.

Some amino acids tend to use rare synonymous codons in SP sequences. We found that SPs with increased rare codon proportion may not directly result in increasing *α*‐amylase secretion, but cell stress was reduced by reducing ROS level. A rise of ROS stress was usually found in cells during recombinant protein production due to imbalance of disulfide bond formation and heterologous protein folding.^[^ ^51^ ^]^ Alleviation of oxidative stress improved cell performances.^[^ ^52^, ^53^ ^]^ Changes in codon bias of SPs may provide benefits for reducing cell burden by adjusting the translation elongation rate.^[^ ^54^ ^]^ Physiological traits of strains varied when using different SPs. Although strains with the top 11 SPs had highest amylase secretion capacities, the substrate consumption rate, metabolites production rate, ATP pool and cellular stress were different among these strains. It was inferred that secretion may be affected by different SPs due to various properties, thereafter resulting in diverse cellular response. This was confirmed by testing several accessory proteins for their influences on protein secretion. For instance, SPs with a basic amino acid on the −2 position suffered more loss in protein secretion in the *SPC1* deletion strain. High hydrophobic SPs increased protein secretion when *SEC72* was deleted. *α*‐Amylase secretion by the SP NCW2 in the *SEC72* deficient strain was 10 times higher than that of the control strain. Sec72 is one of subunits in the Sec62/Sec63 complex.^[^ ^31^, ^32^ ^]^ Cryo‐EM structures in combination with molecular dynamics simulations helps in revealing details of the Sec complex in protein translocation.^[^ ^55^ ^]^ More potential orthogonal components can be identified in future study to precisely control the secretory system in cells. Our study expands understanding of SPs and contribute to both fundamental research and industrial application. These findings can be used to recover cells from SP‐related disorders or design cell factories for efficient protein secretion in the future.

## 4 Experimental Section

### SP Analysis

The genomic ORF sequences of *S. cerevisiae* were downloaded from Saccharomyces Genome Database (SGD, www.yeastgenome.org). ORF containing SPs were identified by SignalP5.0 (https://services.healthtech.dtu.dk/service.php?SignalP‐5.0). NHC parts of SPs were annotated by SignalP3.0. H‐region hydrophobicity compound score was calculated on the Expasy website (https://web.expasy.org/protscale/), using Kyte‐Doolittle hydropathy plotting and window size was set at 9 to identify the most hydrophobic window. The compound score was generated by multiplying the maximum hydrophobic window and H‐region length.^[^ ^56^ ^]^

Sequence conservation analysis was performed on the Weblogo site (https://weblogo.berkeley.edu/logo.cgi) based on same‐length sequences. Sequence Manipulation Suite (SMS, http://www.bioinformatics.org/sms2/codon_usage.html) was used for codon bias analysis.

Subcellular localization of SP‐containing proteins was accessed from the UniProtKB database. Phylogenetic tree analysis was constructed by using the MEGA with the maximum parsimony method. GO terms analysis or enrichment was performed with SGD (https://www.yeastgenome.org/), GOrilla (http://cbl‐gorilla.cs.technion.ac.il/), and Revigo (http://revigo.irb.hr/). All the summarized data are shown in Tables S5–S7 (Supporting Information).

### Strains, Plasmids, and Primers Used in this Study

Expression plasmids were constructed by inserting different fragments in the backbone CPOTud.^[^ ^57^ ^]^ Amylase with different SP expression cassettes were amplified from primers containing SP coding sequences. All the primers are listed in Table S8 (Supporting Information). The *Saccharomyces cerevisiae* strain CEN.PK 530.1CK (*MATa tpi1(41‐707)::loxP*) was used for testing secretion of *α*‐amylase with different SPs. The gene deletion experiment was based on the CRISPR‐cas9 system,^[^ ^58^ ^]^ and performed on the strain MSBP003 (*MATa*, *ura3‐52 can1*△*::cas9‐natNT2 tpi1*△), which was constructed from the parental strain CEN.PK 113.5D (*MATa*, *ura3‐52*).

### Media and Culture Conditions

YPD medium contained 10 g L^−1^ yeast extract, 20 g L^−1^ peptone, and 20 g L^−1^ glucose. YPE medium contained 10 g L^−1^ yeast extract, 20 g L^−1^ peptone, 10 g L^−1^ ethanol, and 0.5 g L^−1^ glucose. For strain 530.1CK, SD‐2 × SCAA medium^[^ ^59^ ^]^ were used for protein expression, which contained 20 g glucose, 6.9 g yeast nitrogen base without amino acids, 1 g BSA, 5.4 g Na_2_HPO_4_, and 8.56 g NaH_2_PO_4_, 0.19 g Arg, 0.4 g Asp, 0.126 g Glu, 0.13 g Gly, 0.14 g His, 0.29 g Ile, 0.4 g Leu, 0.44 g Lys, 0.108 g Met, 0.2 g Phe, 0.22 g Thr, 0.04 g Trp, 0.052 g Tyr, and 0.38 g Val in 1L culture medium (pH = 6.0). For strain MSBP003, SD‐2 × SCAA medium containing 0.04 g L^−1^ uracil were used for protein expression. Yeast strains were cultured in tubes/shake flasks at 30 °C and 200 rpm for 96 h.

### Protein Quantification

For protein quantification, 500 µL cell cultures were centrifuged at 12 000 × g for 1 min for separation of the supernatant and cell pellet. The supernatant was used for extracellular protein measurement. For intracellular protein quantification, the cell pellet was washed with distilled water and resuspended in equal phosphate‐buffered saline (PBS). The cell resuspension was added to a lysing matrix tube for cell lysis and run on a cell homogenizer (Allsheng, Bioprep‐24R) at 6.5 m s^−1^ for 2 min, then cell debris was removed by centrifugation.^[^ ^46^ ^]^ *α*‐Amylase activity was measured by a *α*‐amylase assay kit (Megazyme) and commercial *α*‐amylase (Sigma‐Aldrich, 69.6 U mg^−1^) from *Aspergillus oryzae* was used for quantification as a standard.^[^ ^60^ ^]^ Lipase activity was measured based on a pNP‐palmitate hydrolysis method.^[^ ^61^ ^]^

### Measurements of ROS and ATP

Yeast cells were grown in SD‐2 × SCAA, and samples were taken at the early exponential phase (OD_600_≈1). For ROS measurement, cell pellets from 1 mL broth (12 000 × g, 1 min) were washed with PBS, then 50 mm sodium citrate buffer (SCB) and finally resuspended in 1 mL SCB. 1 µL 50 mm dihydrorhodamine 123 (Cayman) was added and incubated in the dark at room temperature for 30 min. Cell pellets were harvested by centrifugation and washed with SCB and resuspended in equal SCB. Fluorescence was measured with a plate reader (Molecular Devices) with excitation wavelength of 485 nm and emission wavelength of 520 nm. ATP levels were measured by following the assay kit protocol (Solarbio, BC0300).

### Metabolite Measurement

Glucose, ethanol, glycerol, pyruvate, acetate, and succinate were analyzed by an HPLC system (Shimadzu) with loading of cultural supernatant to an Aminex HPX‐87H column (Bio‐Rad). And 5 mm H_2_SO_4_ was used as mobile phase to flow in the HPLC system at a rate of 0.6 mL min^−1^ at 45 °C.^[^ ^59^ ^]^

### Statistical Analysis

All experiments were conducted in duplicates. Data were presented as mean ± SD (standard deviation). SD and *p* values were calculated by using Microsoft Excel 2016 or OriginPro 2019. Statistical significance was defined as *p* < 0.05.

## Conflict of Interest

S.X., X.L., Y.P., C.X., and M.H. have filed a patent application related to part of this work. All other authors declare no competing financial interests.

## Author Contributions

M.H. and S.X. conceived and designed the study. S.X., X.L., Y.P., and C.X. performed experiments. S.X., X.L., Y.P., Y.F., L.Z., M.Z., and M.H. analyzed data. M.Z. and M.H. supervised the study. S.X., Y.P., and M.H. wrote the manuscript. All authors read and agreed with the manuscript.

## Supporting information

Supporting Information

Supporting Information

## Data Availability

The data that support the findings of this study are available in the supplementary material of this article.
