# Comprehensive Analysis of Signal Peptides in *Saccharomyces Cerevisiae* Reveals Features for Efficient Secretion

**DOI:** 10.1002/advs.202300302

**Published:** 2023-02-24

**Authors:** Songlyu Xue, Xiufang Liu, Yuyang Pan, Chufan Xiao, Yunzi Feng, Lin Zheng, Mouming Zhao, Mingtao Huang

*Adv. Sci*. **2023**, *10*, 2203433

DOI: 10.1002/advs.202203433

In the originally published article, Table S8, Supporting Information, contained an error.

Some primer sequences did not match their names in primer lists due to re–sorting in the table. The corrected Table S8 Supporting Information file was published on February 24, 2023, after initial article online publication. The authors state that the correction does not affect the discussion or conclusions and that they sincerely apologize for the unintentional errors.

## Supporting information

Supporting Information

