# Supplementary material for: Comprehensive Analysis of Signal Peptides in Saccharomyces cerevisiae Reveals Features for Efficient Secretion
Source: Adv Sci (Weinh). 2022 Dec 7;10(2):2203433. doi: 10.1002/advs.202203433 (PMC9839866; doi:10.1002/advs.202203433)
Supplement: Supplementary file 1 — Supporting Information [file ADVS-10-2203433-s001.pdf]

ADVANCED SCIENCE
Open Access

Supporting Information

for *Adv. Sci.*, DOI 10.1002/advs.202203433

Comprehensive Analysis of Signal Peptides in *Saccharomyces cerevisiae* Reveals Features for Efficient Secretion

*Songlyu Xue, Xiufang Liu, Yuyang Pan, Chufan Xiao, Yunzi Feng, Lin Zheng, Mouming Zhao and Mingtao Huang**

Supplementary material

Comprehensive analysis of signal peptides in *Saccharomyces cerevisiae* reveals features for efficient secretion

Songlyu Xue[1,2], Xiufang Liu[1,2], Yuyang Pan[1,2], Chufan Xiao[1,2], Yunzi Feng[1,2], Lin Zheng[1,2], Mouming Zhao[1,2], and Mingtao Huang[1,2,*]

[1]School of Food Science and Engineering, South China University of Technology, Guangzhou, 510641, China

[2]Guangdong Food Green Processing and Nutrition Regulation Technologies Research Center, Guangzhou, 510650, China

[*]Correspondence to: huangmt@scut.edu.cn

This file contains:

Supplementary Figure 1-12

Supplementary Table 1-7

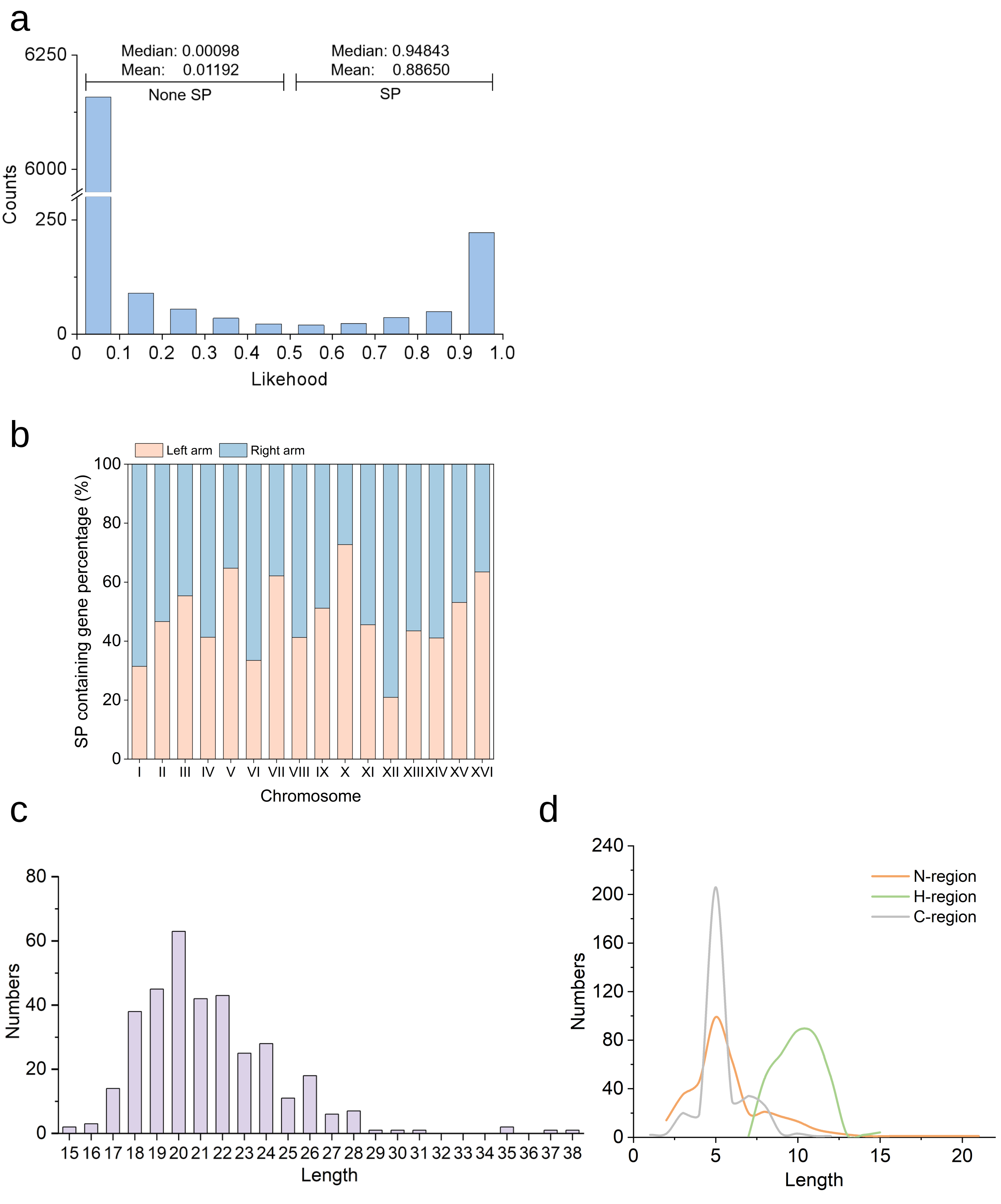

Supplementary Figure 1. Bioinformatic analysis SPs. (a) SP prediction of 6713 ORF sequences by using SignalP5.0. (b) Distribution of 352 predicted SP-containing genes on chromosomes. (c) Length of 352 SP sequences. (d) NHC regions length of 352 SPs.

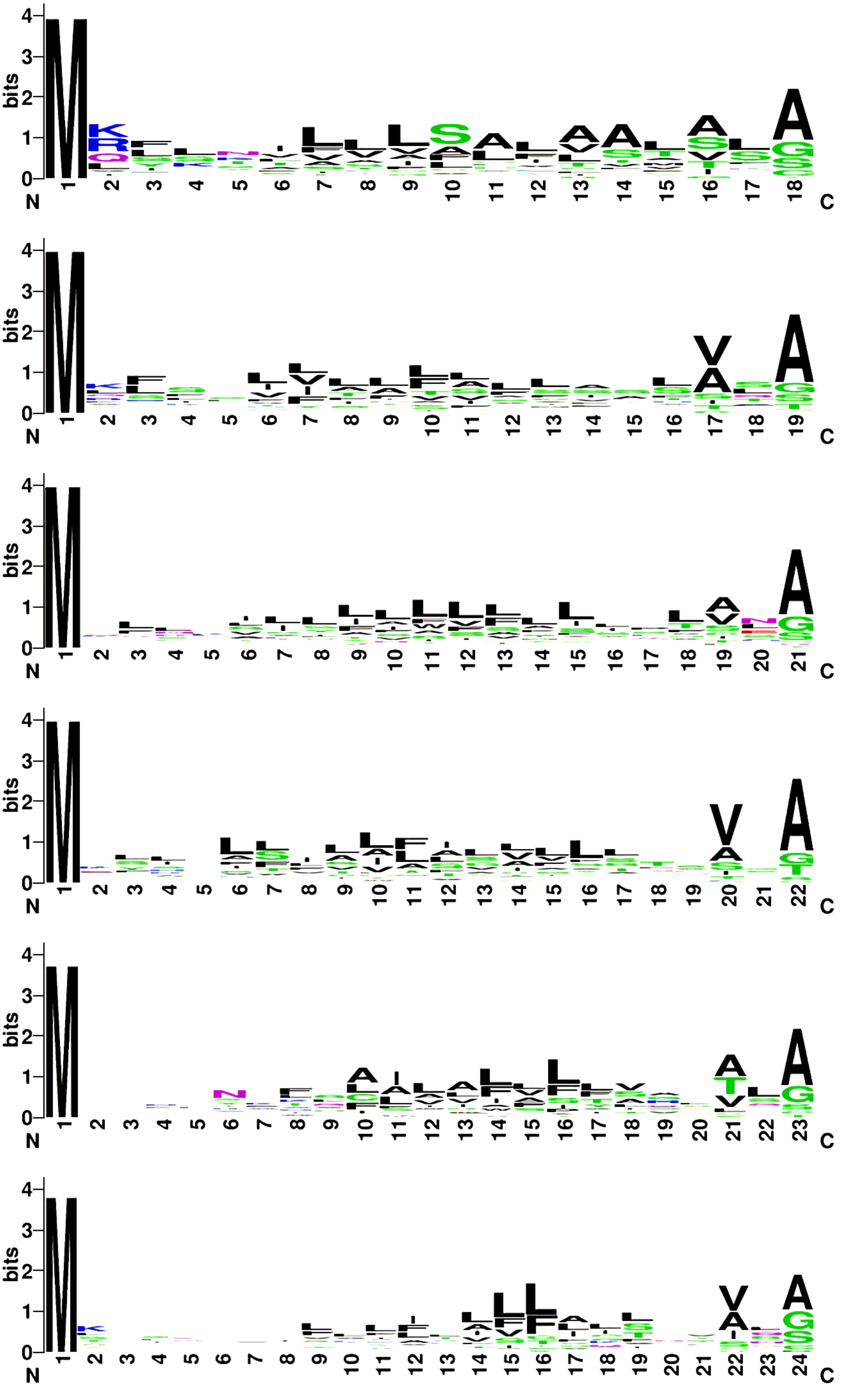

Supplementary Figure 2. Weblogo analysis of SPs with lengths ranging from 18 to 24; SPs with 20 AAs were showed in Fig. 1g.

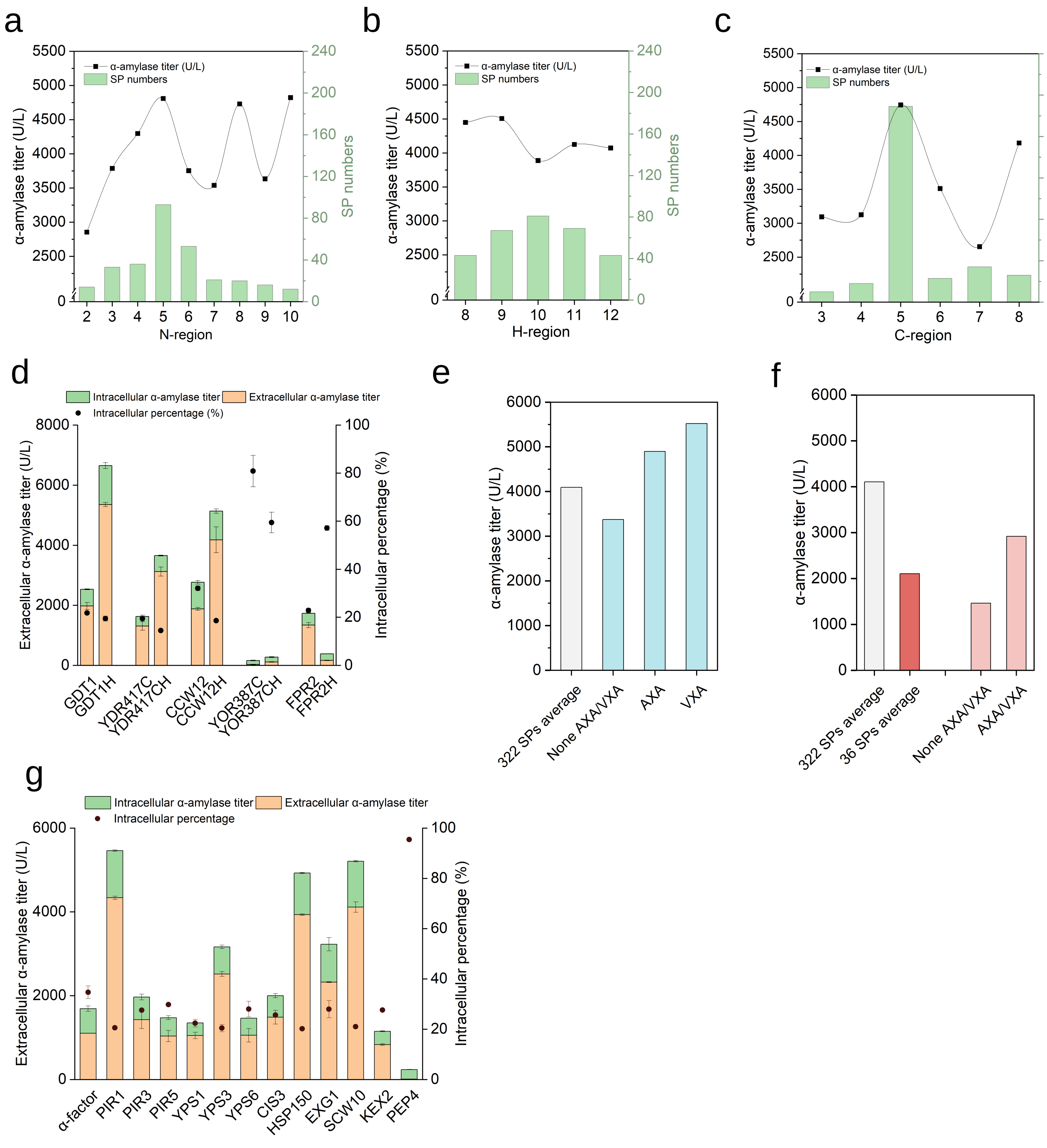

Supplementary Figure 3. α-Amylase secretion led by different SPs were measured. Average secretion level and numbers of SPs with different (a) N-region length, (b) H-region length, and (c) C-region length. (d) Secretion level was altered by changing the H-region hydrophobicity of SPs, which had a hydrophobic compound score of 23 ~25, via amino acid residue deletion. “H” indicated H-region hydrophobic score was changed beyond the interval of 23 ~25. (e) Average of secretory capacity of SPs with AXA or VXA motifs at (-3, -1) position. (f) Average of secretory capacity of 36 SPs with new predicted (-3, -1) motifs fusion with α-amylase cassette. They were divided in different groups according to whether had AXA/VXA motifs or not. (g) α-amylase titer and intracellular percentage of 13 strains using SPs with pre-pro region. Data shown are mean values ±SDs of duplicates.

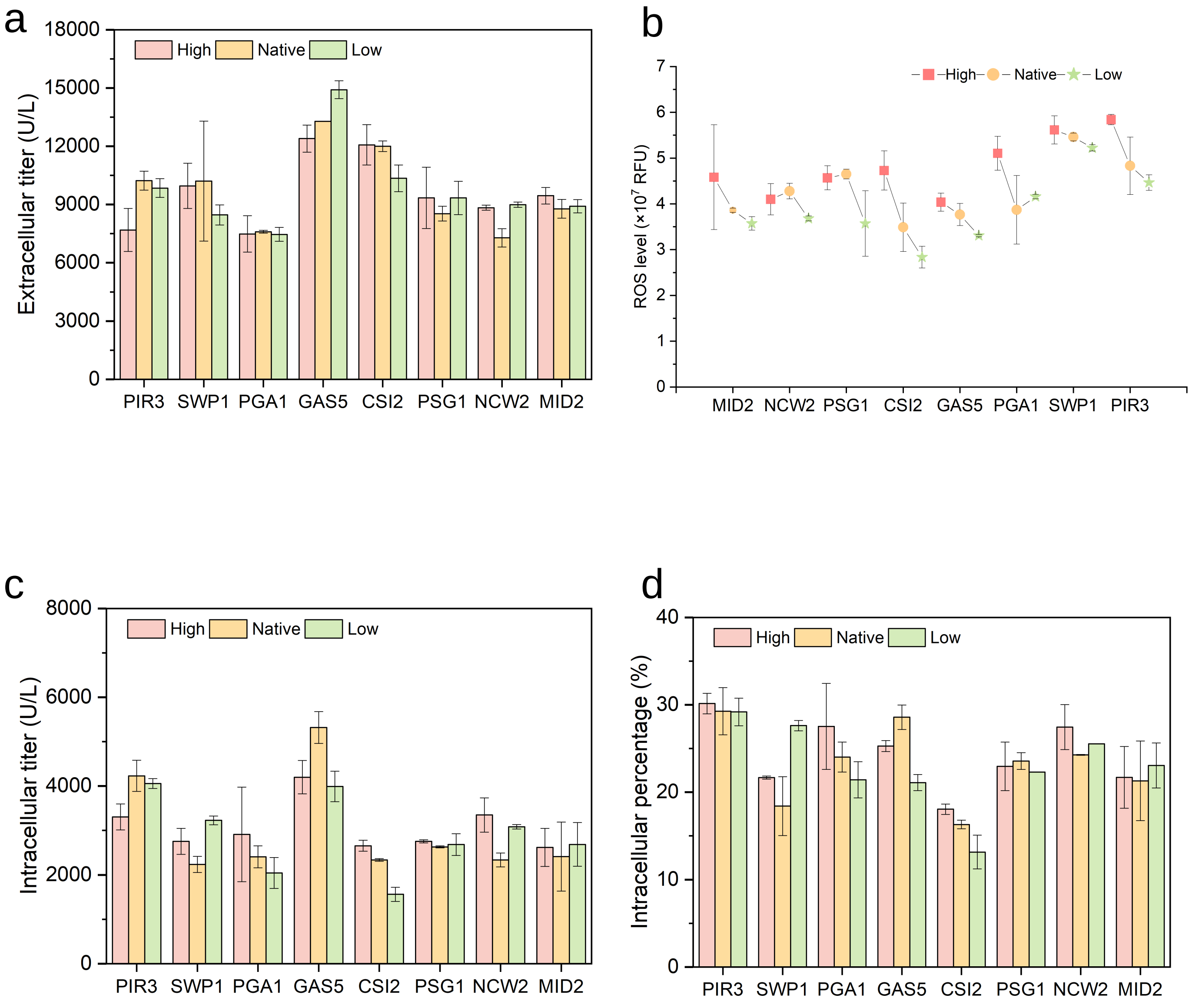

Supplementary Figure 4. α-Amylase secretion by using SPs with different synonymous codon bias in SP sequences. (a) Extracellular α-amylase titer. (b) ROS level of different strains in the exponential phase (OD_{600}≈1). (c) Intracellular α-amylase titer. (d) Intracellular retention percentage. High: SP sequence was changed to high frequency synonymous codon bias. Native: native yeast SP sequence. Low: SP sequence was changed to low frequency synonymous codon bias. Data shown are mean values ±SDs of duplicates.

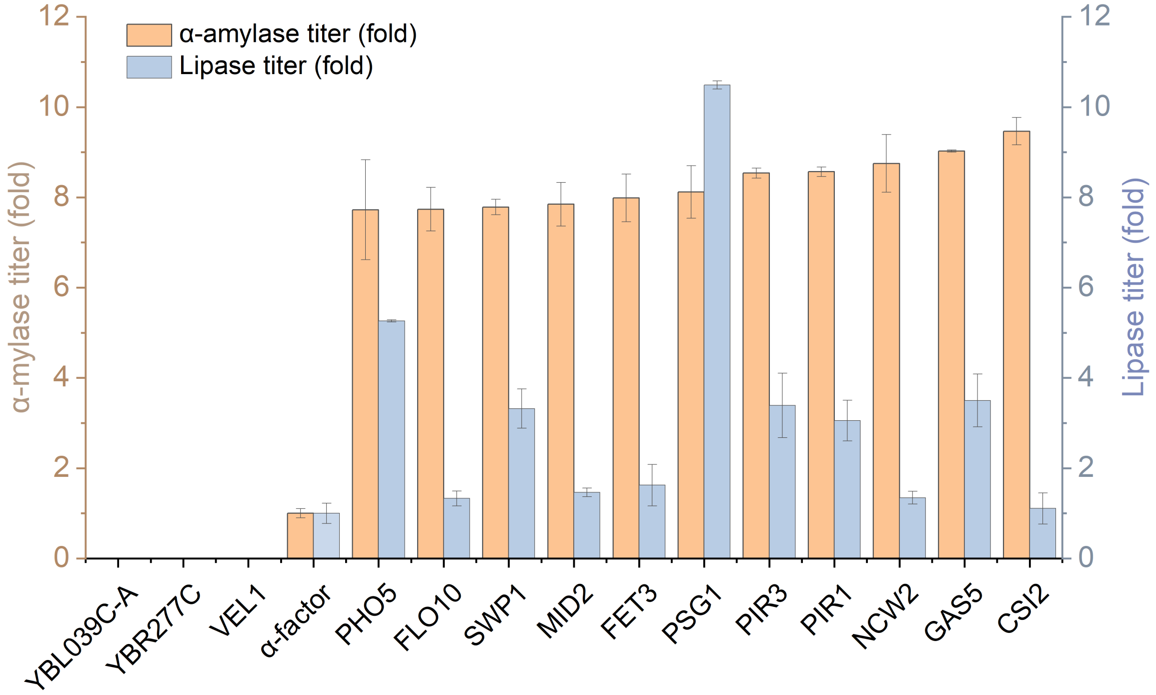

Supplementary Figure 5. Identified super-secreted and poor-secreted SPs were confirmed their secretion capacities by using another protein, lipase. The α-factor leader was used as control. Strains were cultured in SD-2×SCAA medium for 96h at 30℃ and 200rpm. Data shown are mean values ±SDs of duplicates.

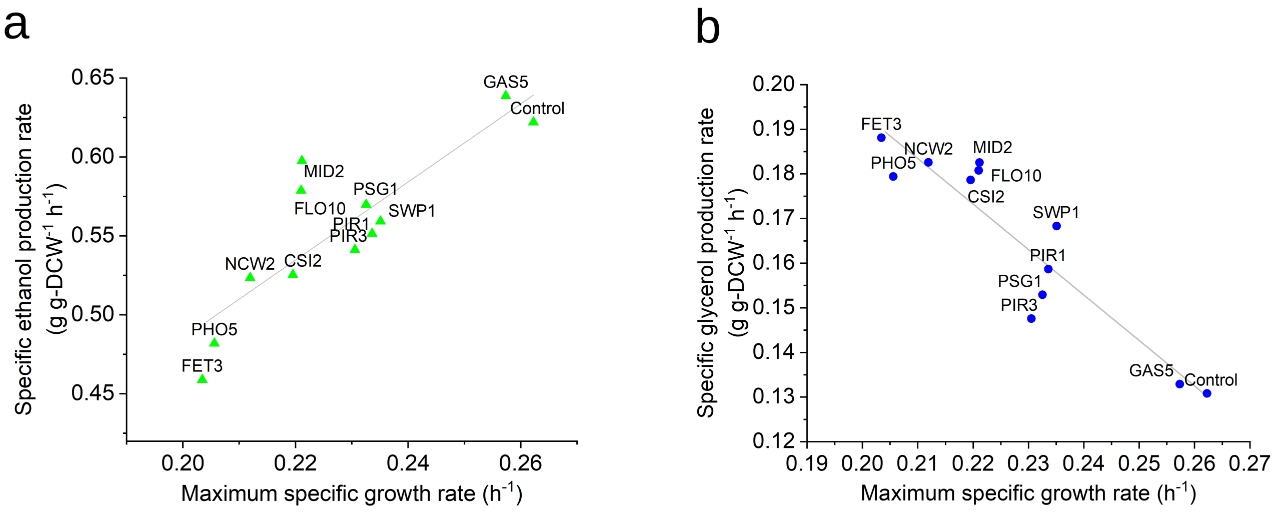

Supplementary Figure 6. Correlation of physiological parameters of strains with 11 super-secreted SPs. Straight lines indicated data fitting. Data shown are mean values ±SDs of duplicates.

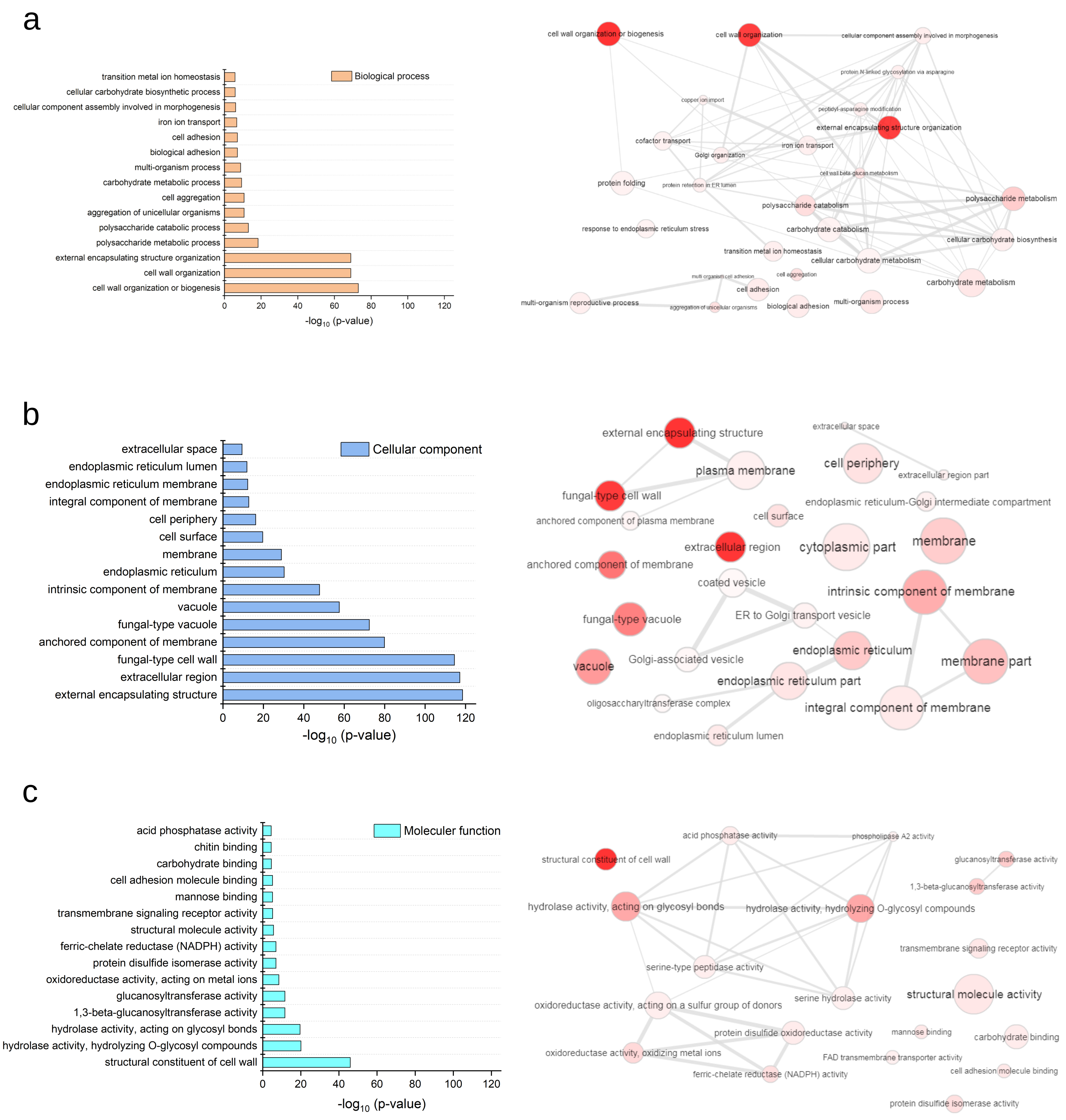

Supplementary Figure 7. GO term enrichment analysis was performed on 322 SP-containing genes. Top15 enriched GO terms are listed in left panels. (a) Biological process. (b) Molecular function. (c) Cellular component.

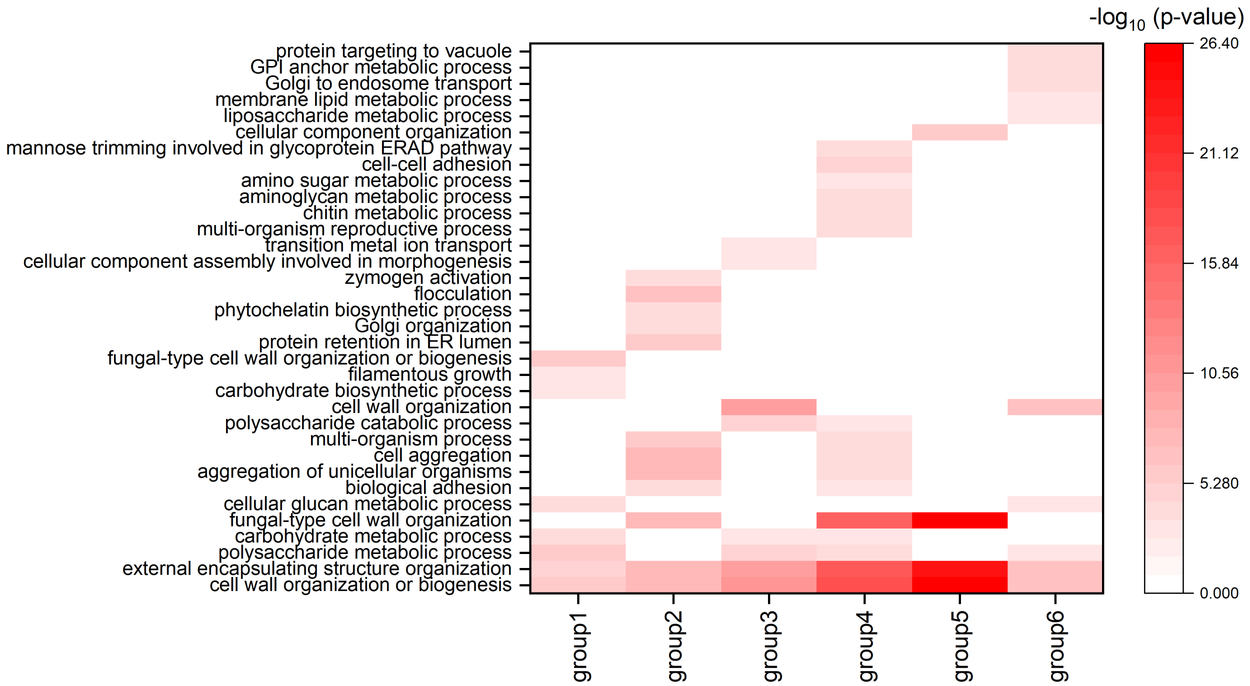

Supplementary Figure 8. GO biological process enrichment of the 6 phylogenetic groups from Fig. 5c.

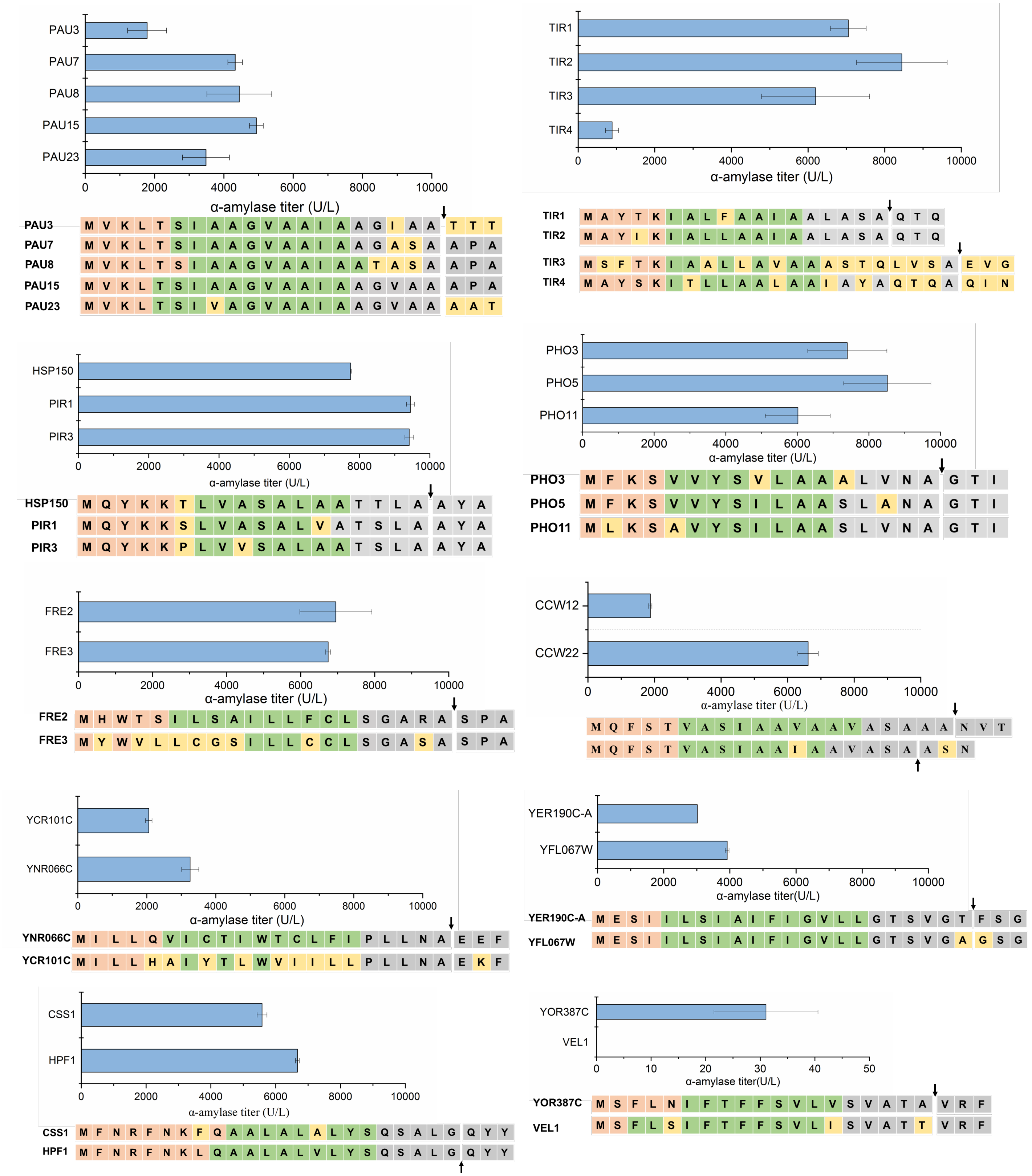

Supplementary Figure 9. Sequence alignment of paralogous SP-containing genes and comparation of α-amylase secretion by these SPs. Data shown are mean values ±SDs of duplicates.

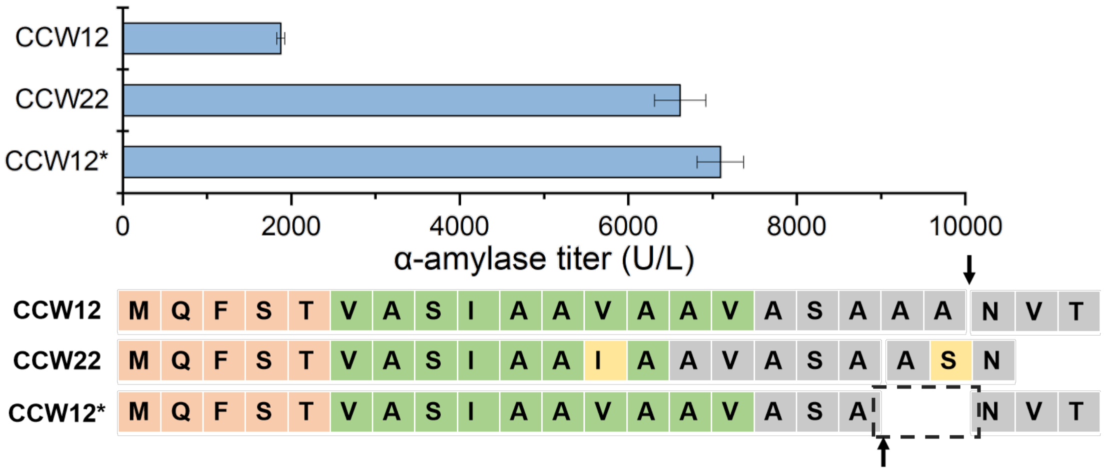

Supplementary Figure 10. Comparation of paralogous SP-containing gene CCW12 and CCW22. CCW12* represents a new sequence by removing the last 2 amino acid residues from CCW12. Data shown are mean values ±SDs of duplicates.

a

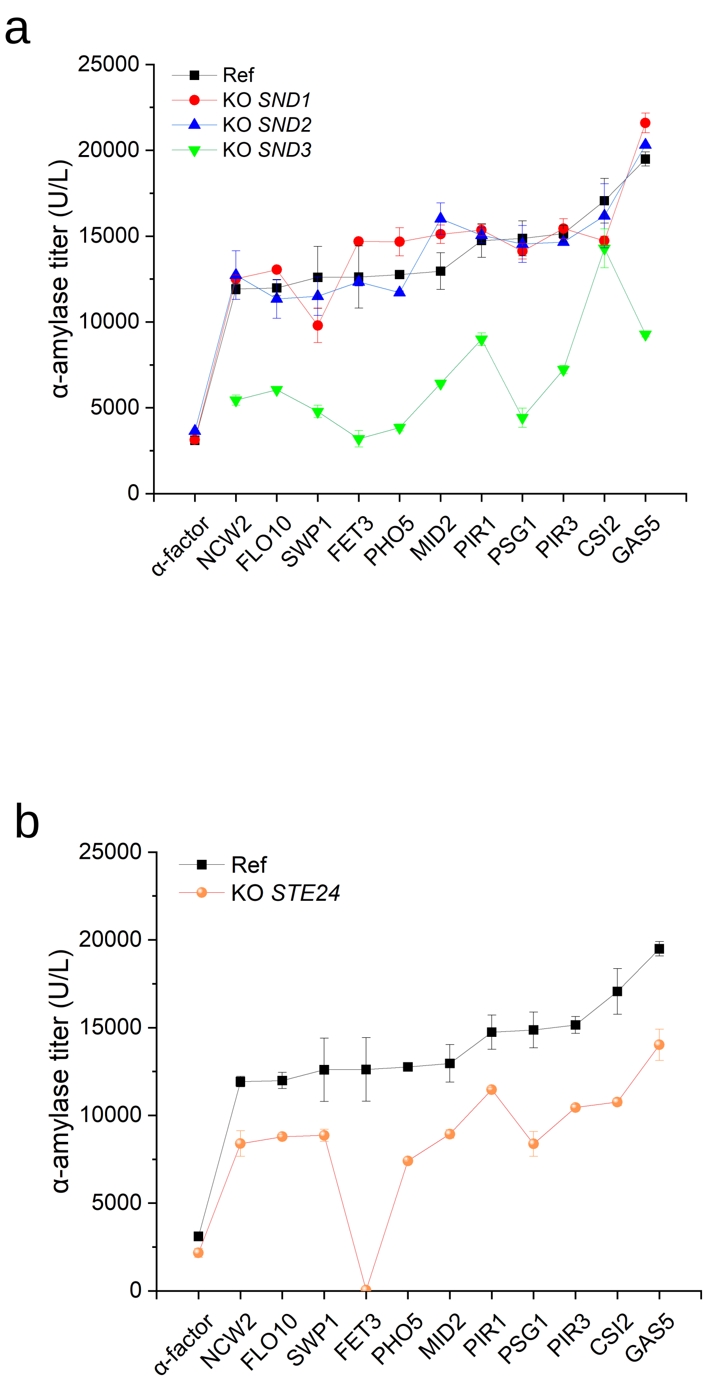

Supplementary Figure 11. α-Amylase secretion level of SPs in different chaperone deletion strains. Ref: strain I58.1CK was used as the reference strain. Data shown are mean values ±SDs of duplicates.

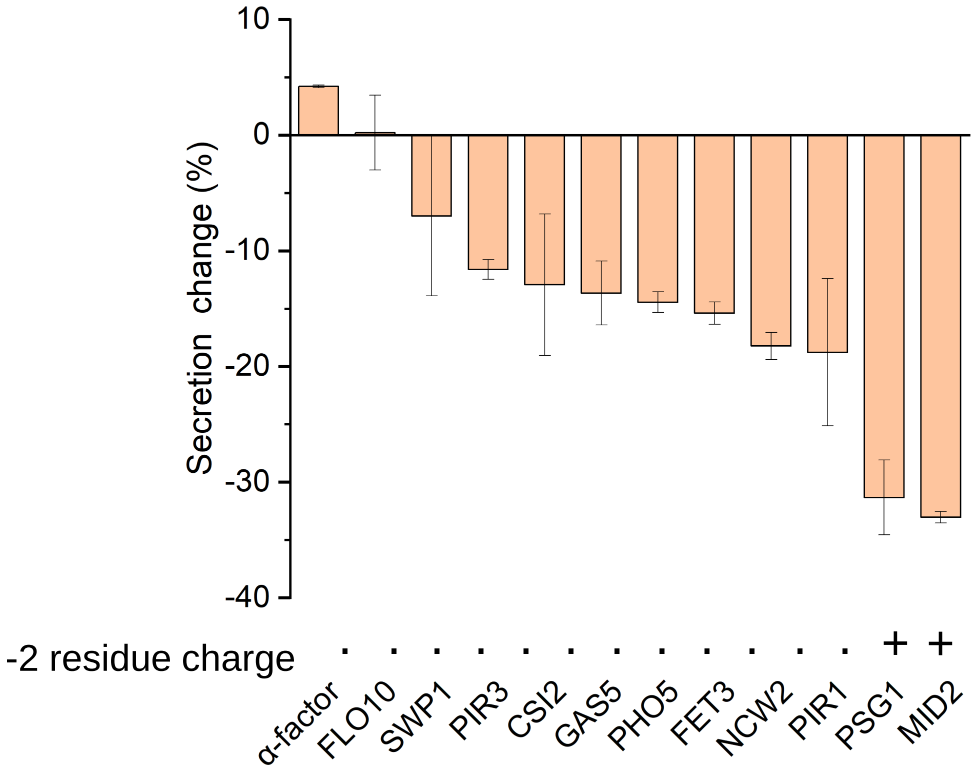

Supplementary Figure 12. α-Amylase secretion decreased in *SPC1* deletion strain with identified 11 super-secreted SPs. The residue charge at the -2 position was shown at the lower edge of the figure. The secretion change was calculated as follow: (amylase secretion by the *SPC1* deletion strain - amylase secretion by the Ref strain) / (amylase secretion by the Ref strain)×100%. Data shown are mean values ±SDs of duplicates.

Table S1 List of SP-containing gene determined by SignalP 5.0

Gene	Gene	Gene	Gene	Gene	Gene	Gene	Gene
YAL007C	YDL010W	YEL042W	YGR106C	YIL162W	YKR058W	YMR008C	YOL052C-
YAL016C-	YDL018C	YEL049W	YGR141W	YIL169C	YKR073C	YMR058W	YOL088C
YAL034C-	YDL024C	YEL053W-	YGR176W	YIL173W	YKR102W	YMR065W	YOL105C
YAL053W	YDL046W	YEL068C	YGR189C	YIL176C	YLL025W	YMR149W	YOL132W
YAL058W	YDL049C	YER011W	YGR279C	YIR019C	YLL051C	YMR158W-	YOL154W
YAL063C	YDL163W	YER038W-	YGR282C	YIR039C	YLL064C	YMR182W-	YOL155C
YAL068C	YDL187C	YER074W-	YGR293C	YIR041W	YLR001C	YMR214W	YOL159C
YAR002C-	YDL237W	YER076C	YGR294W	YJL002C	YLR037C	YMR215W	YOL161C
YAR020C	YDL240C-	YER084W-	YGR296C-	YJL037W	YLR040C	YMR238W	YOR003W
YAR050W	YDR032C	YER087C-	YHL017W	YJL038C	YLR042C	YMR244W	YOR008C
YAR061W	YDR053W	YER137W-	YHL028W	YJL073W	YLR062C	YMR251W-	YOR008C-
YAR066W	YDR055W	YER145C-	YHL037C	YJL078C	YLR083C	YMR272W-	YOR009W
YAR071W	YDR056C	YER150W	YHL046C	YJL079C	YLR084C	YMR297W	YOR010C
YBL008W-	YDR057W	YER188W	YHR057C	YJL116C	YLR104W	YMR305C	YOR016C
YBL017C	YDR077W	YER190C-	YHR069C-	YJL132W	YLR110C	YMR307W	YOR024W
YBL039C-	YDR107C	YFL020C	YHR070C-	YJL158C	YLR120C	YMR325W	YOR030W
YBL096C	YDR134C	YFL041W	YHR078W	YJL159W	YLR121C	YNL003C	YOR085W
YBR013C	YDR144C	YFL048C	YHR079C	YJL160C	YLR155C	YNL012W	YOR139C
YBR067C	YDR221W	YFL051C	YHR101C	YJL171C	YLR157C	YNL019C	YOR149C
YBR078W	YDR236C	YFL067W	YHR110W	YJL174W	YLR158C	YNL024C-	YOR152C
YBR092C	YDR261C	YFR012W	YHR126C	YJL178C	YLR160C	YNL033W	YOR154W
YBR093C	YDR262W	YFR018C	YHR132C	YJL192C	YLR194C	YNL066W	YOR190W
YBR124W	YDR304C	YFR020W	YHR138C	YJL222W	YLR207W	YNL158W	YOR214C
YBR139W	YDR331W	YFR026C	YHR139C	YJL223C	YLR213C	YNL160W	YOR247W
YBR162C	YDR349C	YFR034W-	YHR143W	YJR004C	YLR214W	YNL190W	YOR288C
YBR187W	YDR382W	YFR039C	YHR151C	YJR020W	YLR250W	YNL219C	YOR336W
YBR229C	YDR396W	YFR041C	YHR173C	YJR150C	YLR286C	YNL238W	YOR365C
YBR277C	YDR402C	YGL002W	YHR180W	YJR151C	YLR300W	YNL283C	YOR381W
YBR300C	YDR415C	YGL027C	YHR188C	YJR153W	YLR307W	YNL291C	YOR382W
YBR301W	YDR417C	YGL028C	YHR202W	YKL034W	YLR308W	YNL300W	YOR383C
YCL012C	YDR420W	YGL032C	YHR204W	YKL039W	YLR322W	YNL322C	YOR384W
YCL043C	YDR506C	YGL089C	YHR211W	YKL046C	YLR332W	YNL327W	YOR387C
YCL045C	YDR510C-	YGL138C	YHR212W-	YKL073W	YLR343W	YNL339W-	YOR389W
YCL048W	YDR518W	YGL139W	YHR214W	YKL077W	YLR385C	YNR028W	YPL006W
YCL048W-	YDR519W	YGL200C	YHR215W	YKL096W	YLR390W-	YNR044W	YPL130W
YCL049C	YDR524C-	YGL203C	YIL005W	YKL096W-	YLR413W	YNR060W	YPL154C
YCR011C	YDR534C	YGL228W	YIL011W	YKL162C-	YLR461W	YNR066C	YPL163C
YCR044C	YDR542W	YGL257C	YIL015W	YKL163W	YML012W	YNR067C	YPL187W
YCR045C	YEL001C	YGL258W	YIL023C	YKL164C	YML019W	YNR076W	YPL189W
YCR061W	YEL002C	YGL259W	YIL039W	YKL220C	YML084W	YOL007C	YPL221W
YCR069W	YEL004W	YGL261C	YIL059C	YKL224C	YML128C	YOL011W	YPL283W-
YCR089W	YEL018C-	YGR014W	YIL099W	YKR005C	YML130C	YOL019W	YPR027C
YCR101C	YEL028W	YGR023W	YIL123W	YKR013W	YML133W-	YOL030W	YPR121W
YCR104W	YEL040W	YGR073C	YIL140W	YKR042W	YMR006C	YOL031C	YPR157W

Table S2 List of all codon frequency on SP and ORF-all

AA	CU	SP			ORF-all			AA	CU	SP			ORF-all		
		Num	‰	Frac.	Num	‰	Frac.			Num	‰	Frac.	Num	‰	Frac.
Ala	GCG	86	11.46	0.087	18761	6.20	0.114	Pro	CCG	23	3.07	0.202	16547	5.47	0.125
Ala	GCA	242	32.25	0.244	49165	16.25	0.297	Pro	CCA	44	5.86	0.386	53700	17.75	0.406
Ala	GCT	403	53.71	0.406	60880	20.12	0.368	Pro	CCT	24	3.20	0.211	41141	13.60	0.311
Ala	GCC	261	34.79	0.263	36487	12.06	0.221	Pro	CCC	23	3.07	0.202	21041	6.95	0.159
Cys	TGT	108	14.39	0.543	24645	8.15	0.619	Gln	CAG	53	7.06	0.323	37219	12.30	0.315
Cys	TGC	91	12.13	0.457	15179	5.02	0.381	Gln	CAA	111	14.79	0.677	80930	26.75	0.685
Asp	GAT	21	2.80	0.750	113192	37.41	0.651	Arg	AGG	29	3.87	0.167	28476	9.41	0.212
Asp	GAC	7	0.93	0.250	60663	20.05	0.349	Arg	AGA	63	8.40	0.362	63166	20.88	0.470
								Arg	CGG	13	1.73	0.075	5796	1.92	0.043
Glu	GAG	19	2.53	0.373	58187	19.23	0.300	Arg	CGA	21	2.80	0.121	9707	3.21	0.072
Glu	GAA	32	4.26	0.627	136068	44.97	0.700	Arg	CGT	31	4.13	0.178	19138	6.33	0.142
								Arg	CGC	17	2.27	0.098	8251	2.73	0.061
Phe	TTT	345	45.98	0.578	81147	26.82	0.595								
Phe	TTC	252	33.59	0.422	55144	18.23	0.405	Ser	AGT	102	13.59	0.132	44216	14.61	0.162
								Ser	AGC	82	10.93	0.106	30396	10.05	0.111
Gly	GGG	24	3.20	0.095	18369	6.07	0.123	Ser	TCG	73	9.73	0.095	26683	8.82	0.098
Gly	GGA	63	8.40	0.249	33965	11.23	0.227	Ser	TCA	165	21.99	0.214	58176	19.23	0.213
Gly	GGT	110	14.66	0.435	67509	22.31	0.452	Ser	TCT	211	28.12	0.274	71096	23.50	0.260
Gly	GGC	56	7.46	0.221	29617	9.79	0.198	Ser	TCC	138	18.39	0.179	42850	14.16	0.157
His	CAT	55	7.33	0.611	42231	13.96	0.642	Thr	ACG	56	7.46	0.123	24765	8.19	0.139
His	CAC	35	4.66	0.389	23596	7.80	0.358	Thr	ACA	134	17.86	0.295	54935	18.16	0.308
								Thr	ACT	166	22.12	0.366	60840	20.11	0.341
Ile	ATA	162	21.59	0.280	56172	18.57	0.283	Thr	ACC	98	13.06	0.216	37765	12.48	0.212
Ile	ATT	258	34.39	0.446	91254	30.16	0.459								
Ile	ATC	158	21.06	0.273	51227	16.93	0.258	Val	GTG	126	16.79	0.197	32568	10.76	0.193
								Val	GTA	135	17.99	0.212	37121	12.27	0.220
Lys	AAG	86	11.46	0.358	90758	30.00	0.413	Val	GTT	206	27.46	0.323	64789	21.41	0.385
Lys	AAA	154	20.53	0.642	128853	42.59	0.587	Val	GTC	171	22.79	0.268	33976	11.23	0.202
Leu	TTG	280	37.32	0.212	79746	26.36	0.276	Trp	TGG	87	11.60	1.000	31417	10.38	1.000
Leu	TTA	344	45.85	0.261	80238	26.52	0.277								
Leu	CTG	145	19.33	0.110	32438	10.72	0.112	Tyr	TAT	97	12.93	0.577	58293	19.27	0.570
Leu	CTA	199	26.52	0.151	40813	13.49	0.141	Tyr	TAC	71	9.46	0.423	43899	14.51	0.430
Leu	CTT	214	28.52	0.162	38481	12.72	0.133								
Leu	CTC	137	18.26	0.104	17506	5.79	0.061	End	TGA	0	0.00	0.000	2126	0.70	0.311
								End	TAG	0	0.00	0.000	1558	0.51	0.228
Met	ATG	430	57.31	1.000	63108	20.86	1.000	End	TAA	0	0.00	0.000	3163	1.05	0.462
Asn	AAT	101	13.46	0.647	110229	36.43	0.598								
Asn	AAC	55	7.33	0.353	74169	24.51	0.402								

AA: Amino acid; CU: Codon Usage; Num: Number; ‰: Occurrences per thousand codons; Frac: Fraction of synonymous codons; SP: Signal Peptide; ORF-all: genome-wide open reading frame

Table S3 GO enrichment analysis of SP-containing genes on different chromosomes

Chr	Process	Function	Component
I	Flocculation<br>protein retention in ER lumen<br>cell aggregation<br>Golgi organization	carbohydrate binding<br>mannose binding	fungal-type cell wall<br>external encapsulating structure<br>anchored component of membrane<br>endoplasmic reticulum-Golgi intermediate compartment<br>extracellular region<br>COPII-coated ER to Golgi transport vesicle
II		acid phosphatase activity	extracellular region<br>fungal-type cell wall<br>external encapsulating structure
III			fungal-type cell wall<br>external encapsulating structure<br>anchored component of membrane<br>intrinsic component of membrane<br>membrane
IV	fungal-type cell wall organization<br>external encapsulating structure organization<br>cell wall organization or biogenesis<br>polysaccharide metabolic process		extracellular region<br>external encapsulating structure<br>cell wall<br>anchored component of membrane<br>fungal-type vacuole<br>endoplasmic reticulum lumen<br>vacuole<br>endoplasmic reticulum<br>endoplasmic reticulum part
V	fungal-type cell wall organization<br>cell wall chitin metabolic process<br>external encapsulating structure organization	structural constituent of cell wall	intrinsic component of membrane<br>fungal-type cell wall<br>membrane part<br>external encapsulating structure<br>endoplasmic reticulum<br>anchored component of membrane<br>endoplasmic reticulum membrane<br>membrane
VI			endoplasmic reticulum
VII	cell wall organization or biogenesis<br>fungal-type cell wall organization or biogenesis<br>external encapsulating structure organization<br>multi-organism reproductive process	hydrolase activity, hydrolyzing O-glycosyl compounds<br>hydrolase activity, acting on glycosyl bonds<br>glucanosyltransferase activity	cell surface<br>extracellular region<br>fungal-type cell wall<br>external encapsulating structure<br>vacuole<br>endoplasmic reticulum<br>fungal-type vacuole<br>intrinsic component of membrane

	multi-organism process	1,3-beta-glucanosyltransferase activity	
VII	cell wall organization<br>external encapsulating structure organization<br>cell wall organization or biogenesis		fungal-type vacuole<br>vacuole<br>external encapsulating structure<br>cell wall<br>anchored component of membrane<br>intrinsic component of membrane
IX	polysaccharide catabolic process<br>fungal-type cell wall organization<br>external encapsulating structure organization<br>cell wall organization or biogenesis<br>macromolecule catabolic process<br>carbohydrate catabolic process	hydrolase activity, acting on glycosyl bonds	extracellular region<br>external encapsulating structure<br>cell wall<br>fungal-type vacuole<br>vacuole<br>anchored component of membrane
X	external encapsulating structure organization<br>cell wall organization or biogenesis<br>fungal-type cell wall organization<br>sterol transport<br>organic hydroxy compound transport	structural constituent of cell wall<br>structural molecule activity	external encapsulating structure<br>cell wall<br>extracellular region<br>fungal-type vacuole<br>vacuole<br>anchored component of membrane<br>extracellular space<br>extracellular region part<br>intrinsic component of membrane<br>membrane part
XI	fungal-type cell wall organization or biogenesis<br>external encapsulating structure organization<br>cell wall organization or biogenesis	structural constituent of cell wall	extracellular region<br>fungal-type cell wall<br>external encapsulating structure<br>anchored component of membrane<br>fungal-type vacuole
XII	fungal-type cell wall organization<br>external encapsulating structure organization<br>cell wall organization or biogenesis<br>polysaccharide catabolic process	structural constituent of cell wall<br>chitin deacetylase activity<br>hydrolase activity, acting on carbon-nitrogen (but not peptide) bonds	fungal-type cell wall<br>external encapsulating structure<br>fungal-type vacuole<br>vacuole<br>extracellular region<br>anchored component of membrane<br>anchored component of plasma membrane

	chitin catabolic process<br>polysaccharide metabolic process<br>aminoglycan metabolic process<br>amino sugar metabolic process<br>organonitrogen compound catabolic process<br>carbohydrate derivative catabolic process<br>macromolecule catabolic process<br>catabolic process<br>carbohydrate metabolic process	ferric-chelate reductase (NADPH) activity<br>hydrolase activity, acting on carbon-nitrogen (but not peptide) bonds, in linear amides	intrinsic component of membrane<br>membrane part<br>plasma membrane<br>plasma membrane part<br>membrane
XIII	cell wall (1->3)-beta-D-glucan biosynthetic process<br>protein folding in endoplasmic reticulum<br>phospholipid catabolic process	glucanosyltransferase activity<br>1,3-beta-glucanosyltransferase activity<br>lysophospholipase activity	endoplasmic reticulum<br>anchored component of membrane<br>fungal-type vacuole<br>extracellular region<br>membrane<br>plasma membrane<br>intrinsic component of membrane<br>vacuole<br>membrane part<br>oligosaccharyltransferase complex<br>fungal-type cell wall<br>external encapsulating structure
XIV		hydrolase activity, hydrolyzing O-glycosyl compounds	extracellular region<br>fungal-type cell wall<br>external encapsulating structure<br>intrinsic component of membrane<br>fungal-type vacuole<br>membrane part<br>anchored component of membrane<br>vacuole<br>membrane<br>integral component of membrane<br>cell septum
XV	siderophore transport<br>iron ion homeostasis<br>cell wall organization or biogenesis<br>cell wall organization	disulfide oxidoreductase activity<br>ferric-chelate reductase (NADPH) activity<br>protein disulfide isomerase activity	fungal-type vacuole<br>extracellular region<br>vacuole<br>fungal-type cell wall<br>external encapsulating structure<br>anchored component of membrane

	protein N-linked glycosylation via asparagine<br>external encapsulating structure organization<br>iron coordination entity transport<br>peptidyl-asparagine modification<br>fungal-type cell wall beta-glucan biosynthetic process	intrinsic component of membrane<br>cell periphery<br>membrane part<br>membrane<br>endoplasmic reticulum lumen<br>cell surface<br>endoplasmic reticulum<br>cytoplasmic part
XVI	membrane lipid metabolic process	vacuole<br>extracellular region<br>fungal-type vacuole

Table S4 H-region hydrophobicity of SPs

Signal peptide	H-region	sum	average
PIR1	SLVASALV	17.69	1.25
PIR3	PLVVSALAA	19.90	1.33
PSG1	ILIFFSLASLY	27.38	1.42
GAS5	LTSAFVLSA	18.80	1.45
PHO5	VVYSILAA	15.46	1.58
CSI2	VILLLHLFAL	28.11	1.75
NCW2	CSILFTTLI	21.10	1.81
α-factor	IFTAVLFAA	22.80	1.87
FLO10	IFLTGLFLLSVA	32.27	1.87
SWP1	LAALVSCI	21.51	2.08
FET3	LLSIAVLLFSML	36.94	2.29
MID2	LLLLILSCI	29.70	2.30

H-region hydrophobic scores of 12 SPs were generated by using Kyte-Doolittle hydropathy plotting with window size was set at 9. The average is the sum of all the amino acids hydrophobicity divided by H-region length.

Table S5 GO Term biological process analysis of SP-containing genes

Term ID	Name	Frequency	Value
GO:0071554	cell wall organization or biogenesis	4.69%	-73.0013
GO:0071555	cell wall organization	3.80%	-68.9281
GO:0045229	external encapsulating structure organization	3.80%	-68.9281
GO:0005976	polysaccharide metabolic process	1.04%	-18.3298
GO:0000272	polysaccharide catabolic process	0.27%	-13.0329
GO:0098630	aggregation of unicellular organisms	0.08%	-10.7235
GO:0098743	cell aggregation	0.08%	-10.7235
GO:0005975	carbohydrate metabolic process	3.86%	-9.3036
GO:0051704	multi-organism process	2.77%	-8.752
GO:0022610	biological adhesion	0.28%	-7.0372
GO:0007155	cell adhesion	0.28%	-7.0372
GO:0006826	iron ion transport	0.65%	-6.6946
GO:0010927	cellular component assembly involved in morphogenesis	1.13%	-6.0768
GO:0034637	cellular carbohydrate biosynthetic process	0.76%	-5.8477
GO:0055076	transition metal ion homeostasis	1.54%	-5.7747
GO:0018279	protein N-linked glycosylation via asparagine	0.10%	-5.7282
GO:0018196	peptidyl-asparagine modification	0.10%	-5.7282
GO:0007030	Golgi organization	0.56%	-5.7077
GO:0006621	protein retention in ER lumen	0.17%	-5.5436
GO:0044703	multi-organism reproductive process	2.75%	-5.5317
GO:0016051	carbohydrate biosynthetic process	1.03%	-5.2684
GO:0044036	cell wall macromolecule metabolic process	0.56%	-5.1141
GO:0006457	protein folding	1.97%	-4.6946
GO:0034976	response to endoplasmic reticulum stress	1.31%	-4.3382
GO:0006643	membrane lipid metabolic process	1.29%	-3.8996
GO:0046467	membrane lipid biosynthetic process	1.03%	-3.6904
GO:0016043	cellular component organization	31.99%	-3.6144
GO:0006030	chitin metabolic process	0.45%	-3.5272
GO:0006022	aminoglycan metabolic process	0.45%	-3.5272
GO:0070085	glycosylation	1.49%	-3.5114
GO:0046475	glycerophospholipid catabolic process	0.13%	-3.4134
GO:0030001	metal ion transport	1.61%	-3.4123
GO:0070589	cellular component macromolecule biosynthetic process	0.56%	-3.3862
GO:0034975	protein folding in endoplasmic reticulum	0.23%	-3.3516
GO:0035350	FAD transmembrane transport	0.07%	-3.3036
GO:0015883	FAD transport	0.07%	-3.3036
GO:1903509	liposaccharide metabolic process	0.63%	-3.2899
GO:0006040	amino sugar metabolic process	0.50%	-3.2248

GO:0000413	protein peptidyl-prolyl isomerization	0.27%	-3.0496

Table S6 GO Term molecular function analysis of SP-containing genes

Term ID	Name	Frequency	Value
GO:0005199	structural constituent of cell wall	0.75%	-45.9788
GO:0004553	hydrolase activity, hydrolyzing O-glycosyl compounds	0.81%	-20.0675
GO:0016798	hydrolase activity, acting on glycosyl bonds	1.00%	-19.6615
GO:0042124	1,3-beta-glucanosyltransferase activity	0.15%	-11.7033
GO:0042123	glucanosyltransferase activity	0.15%	-11.7033
GO:0016722	oxidoreductase activity, acting on metal ions	0.22%	-8.5017
GO:0003756	protein disulfide isomerase activity	0.12%	-6.9626
GO:0052851	ferric-chelate reductase (NADPH) activity	0.12%	-6.9626
GO:0005198	structural molecule activity	5.79%	-5.7011
GO:0004888	transmembrane signaling receptor activity	0.33%	-5.2197
GO:0005537	mannose binding	0.12%	-5.2027
GO:0050839	cell adhesion molecule binding	0.07%	-5.1878
GO:0030246	carbohydrate binding	0.56%	-4.5884
GO:0008061	chitin binding	0.08%	-4.5058
GO:0003993	acid phosphatase activity	0.15%	-4.4609
GO:0015035	protein-disulfide reductase activity	0.40%	-4.2967
GO:0016667	oxidoreductase activity, acting on a sulfur group of donors	0.61%	-4.06
GO:0008236	serine-type peptidase activity	0.28%	-3.9031
GO:0016860	intramolecular oxidoreductase activity	0.32%	-3.6021
GO:0017171	serine hydrolase activity	0.33%	-3.4647
GO:0016018	cyclosporin A binding	0.13%	-3.4134
GO:0015230	FAD transmembrane transporter activity	0.07%	-3.3036
GO:0102545	phosphatidyl phospholipase B activity	0.07%	-3.3036

Table S7 GO Term cellular component analysis of SP-containing genes

Term ID	Name	Frequency	Value
GO:0030312	external encapsulating structure	2.29%	-118.5719
GO:0005576	extracellular region	1.96%	-117.1681
GO:0009277	fungal-type cell wall	2.17%	-114.5391
GO:0031225	anchored component of membrane	1.08%	-79.9031
GO:0000324	fungal-type vacuole	7.64%	-72.4498
GO:0005773	vacuole	8.58%	-57.6003
GO:0031224	intrinsic component of membrane	22.07%	-47.8297
GO:0005783	endoplasmic reticulum	11.42%	-30.3458
GO:0016020	membrane	34.69%	-28.9172
GO:0009986	cell surface	0.30%	-19.6778
GO:0071944	cell periphery	13.73%	-16.1986
GO:0016021	integral component of membrane	20.92%	-12.7959
GO:0005789	endoplasmic reticulum membrane	6.49%	-12.3028
GO:0005788	endoplasmic reticulum lumen	0.33%	-11.9393
GO:0005615	extracellular space	0.15%	-9.4634
GO:0005793	endoplasmic reticulum-Golgi intermediate compartment	0.41%	-8.5287
GO:0005886	plasma membrane	8.95%	-8.2161
GO:0030134	COPII-coated ER to Golgi transport vesicle	0.75%	-7.5243
GO:0046658	anchored component of plasma membrane	0.10%	-5.7282
GO:0005798	Golgi-associated vesicle	0.65%	-4.9914
GO:0008250	oligosaccharyltransferase complex	0.15%	-4.4609
GO:0030135	coated vesicle	1.72%	-4.4413
GO:0031226	intrinsic component of plasma membrane	1.04%	-3.9469
GO:0005794	Golgi apparatus	4.85%	-3.2573
